# Enhancing parasitic organism detection in microscopy images through deep learning and fine-tuned optimizer

**DOI:** 10.1038/s41598-024-56323-8

**Published:** 2024-03-08

**Authors:** Yogesh Kumar, Pertik Garg, Manu Raj Moudgil, Rupinder Singh, Marcin Woźniak, Jana Shafi, Muhammad Fazal Ijaz

**Affiliations:** 1 Department of CSE, School of Technology, Pandit Deendayal Energy University, Gandhinagar, Gujarat India; 2 Department of CSE, Swami Vivekanand Institute of Engineering and Technology, Ramnagar, India; 3 Department of Computer Science & Engineering, Bhai Gurdas Institute of Engineering & Technology, Sangrur, Punjab India; 4 Chitkara University Institute of Engineering and Technology, Chitkara University, Rajpura, Punjab India; 5 Faculty of Applied Mathematics, Silesian University of Technology, Kaszubska 23, 44100 Gliwice, Poland; 6 Department of Computer Engineering and Information, College of Engineering in Wadi Al Dawasir, Prince Sattam Bin Abdulaziz University, 11991 Wadi Al Dawasir, Saudi Arabia; 7 School of IT and Engineering, Melbourne Institute of Technology, Melbourne, 3000 Australia

**Keywords:** Parasitic organisms, Segmentation, Watershed, Deep learning, Optimizers, Adam, InceptionV3, InceptionResNetV2, Computer science, Mathematics and computing, Information technology

## Abstract

Parasitic organisms pose a major global health threat, mainly in regions that lack advanced medical facilities. Early and accurate detection of parasitic organisms is vital to saving lives. Deep learning models have uplifted the medical sector by providing promising results in diagnosing, detecting, and classifying diseases. This paper explores the role of deep learning techniques in detecting and classifying various parasitic organisms. The research works on a dataset consisting of 34,298 samples of parasites such as Toxoplasma Gondii, Trypanosome, Plasmodium, Leishmania, Babesia, and Trichomonad along with host cells like red blood cells and white blood cells. These images are initially converted from RGB to grayscale followed by the computation of morphological features such as perimeter, height, area, and width. Later, Otsu thresholding and watershed techniques are applied to differentiate foreground from background and create markers on the images for the identification of regions of interest. Deep transfer learning models such as VGG19, InceptionV3, ResNet50V2, ResNet152V2, EfficientNetB3, EfficientNetB0, MobileNetV2, Xception, DenseNet169, and a hybrid model, InceptionResNetV2, are employed. The parameters of these models are fine-tuned using three optimizers: SGD, RMSprop, and Adam. Experimental results reveal that when RMSprop is applied, VGG19, InceptionV3, and EfficientNetB0 achieve the highest accuracy of 99.1% with a loss of 0.09. Similarly, using the SGD optimizer, InceptionV3 performs exceptionally well, achieving the highest accuracy of 99.91% with a loss of 0.98. Finally, applying the Adam optimizer, InceptionResNetV2 excels, achieving the highest accuracy of 99.96% with a loss of 0.13, outperforming other optimizers. The findings of this research signify that using deep learning models coupled with image processing methods generates a highly accurate and efficient way to detect and classify parasitic organisms.

## Introduction

Parasites are the different groups of organisms that are present at every corner of our house. From the very early times, they have played a very important role in shaping the course of human history, biology, as well as our immune system^1^. In fact, such complex interplay between humans and parasites has many implications that extend far beyond the mere infections caused by parasites. Parasites, from microscopic protozoa to complex multicellular helminths, have astonishing life strategies that challenge the understanding of the relationship between evolution and biology^2^. Parasite diseases are caused by various organisms such as ectoparasites, protozoa, etc., and throw very detrimental as well as severe health issues on the human being as they are long-lasting and often show consequences that are life-threatening if not treated at the right time. Parasite diseases affect millions of people at every nook of the globe and thereby it is categorized as a significant global health challenge^3^.

Traditionally, diagnosing parasitic diseases is laborious and time-consuming as it includes serological tests, molecular techniques, and microscopy. No doubt these methods have proven to be effective but at the same time they also demand highly skilled and professional people who can understand and analyze the disease properly. Thereby, it is important to have an early detection of such infections which is crucial for timely intervention and effective treatment^4^. In these years, the application of machine and deep learning models has shown exciting results in the medical sector by improving and enhancing the precision and efficiency of detecting and diagnosing multiple threatened diseases. Likewise, these techniques have also offered and exciting possibilities by improving the precision of detecting and classifying parasitic diseases^5^.

Machine learning techniques such as support vector machine, random forest, decision trees, etc., and deep learning techniques like RNN, CNN, etc. are very much capable of recognizing the patterns and classifying the given data. These techniques have played an amazing role in analyzing medical data which includes tissue samples, blood smears, and diagnostic images which can work on the accuracy of detecting and classifying various parasite diseases^6^. In addition to this, the ML and Dl techniques are not restricted to the aforementioned traditional diagnostic methods. In fact, they can be also applied to the analysis of data from emerging technologies, such as genomic sequencing, rapid diagnostic tests, and mobile health applications, making the diagnostic process faster and more accessible^7^.

Therefore, this study aims to investigate the usage of machine learning and deep learning methodologies in the identification and categorization of diverse parasitic diseases. By thoroughly examining existing research in this field, we will proceed to develop a system that incorporates multiple deep-learning models and fine-tune their parameters by using various optimization techniques to obtain the optimized results. The implementation of this approach is probable to introduce innovative concepts and improvements to the field of parasite detection.

The contribution made to conduct the research is hereby presented as follows:

- Exploring the work done by the researchers in the field of detecting and classifying various parasitic organisms.
- Compilation of a diverse dataset containing 34,298 samples encompassing various parasitic organisms including Plasmodium, Toxoplasma Gondii (T.gondii), Babesia, Leishmania, Trypanosome, and Trichomonad. Inclusion of host cells such as red blood cells and white blood cells, enhancing the dataset's complexity and real-world relevance.
- Conversion of images from RGB to grayscale and extraction of morphological features, such as area, perimeter, height, and width, facilitating a detailed understanding of the image characteristics.
- Implementation of Otsu thresholding and watershed techniques to distinguish foreground from background, ensuring accurate identification of regions of interest.
- Using different deep transfer learning models including VGG19, InceptionV3, ResNet50V2, ResNet152V2, EfficientNetB3, EfficientNetB0, MobileNetV2, Xception, DenseNet169, and the hybrid model InceptionResNetV2.
- Fine-tuning of model parameters using three different optimizer techniques: RMSprop, SGD, and Adam.
- Thorough examination and comparison of these models on the basis of various performance metrics to demonstrate their efficacy in parasitic organism classification.

## Related work

A lot of contributions have been made by researchers for the detection and classification of parasitic organisms. Zhang et al.^8^ explored the effectiveness of deep learning models in diagnosing infectious and parasitic diseases caused by protozoan parasites. They discussed the limitations of traditional microscopic examination methods and highlighted how deep learning models have shown exceptional performance in improving disease diagnosis. This research underscores the transformative potential of artificial intelligence in healthcare, especially in addressing infectious diseases, and suggests a promising future for deep learning in advancing global public health efforts. Alharbi et al.^9^ worked on the development of a model that could increase its robustness as well as precision so that it could effectively distinguish between the uninfected blood cells and parasitic cells. The work was carried out on the dataset of 13,750 parasitized and uninfected samples and was applied to the neural network, XGBoost and SVM model. During experimentation, it was found that the best accuracy to differentiate between parasite cells from healthy ones had been computed by the SVM model with 94% as compared to the other three models. Additionally, the XGBoost model also did well by obtaining 90% accuracy but the neural network lacked by obtaining only 80%. Researchers also applied the CNN model and it was found that the model boosted the accuracy level with 97% for the same sample. Wang et al.^10^ applied object detection techniques such as a Single shot multibox detector and an Incremental Improvement version of You Only Look Once to recognize leukocytes. The dataset of 14,700 annotated images was used and tested the model by using 1120 labeled images and 7868 labeled single object images to represent 11 types of peripheral leukocytes. The researchers conducted their work on NVIDIA GTX1080Ti GPU where the model obtained 90.09% accuracy by investing 53 ms in each image. Leng et al.^11^ mentioned a pure transformer based on an end-to-end object detection network which was based on DETR to identify leukocytes. The pyramid vision transformer and deformable attention module were added to the DETR model to boost the performance and convergence speed. Two types of the dataset were used by the researchers one was the Common Objects in Context dataset to obtain the pre-trained weights and another Raabin Leukocyte dataset which was used to train the transfer learning model. While execution it was found that the upgraded DETR performed quite well than the CNN and original DETR with the mean accuracy of 96.1%. Li et al.^12^ examined the performance of the deep learning model to automatically detect leukocytes. A novel dataset was created by the researchers of 6273 images which included 8595 leukocytes and nine clinical interference variables. Six detection techniques were trained with this dataset and later were presented as a robust ensemble model. On examining the model with the test dataset, it was found that it computed 0.922 mean of average recall, 0.853 mean of average precision, and an accuracy of 98.84%. Furthermore, the authors examined the test results of several models and discovered multiple identical false detections of the models. They then made appropriate clinic recommendations. Gonçalves et al.^13^ detected VL in humans by applying deep learning algorithms to the slide images of the bone marrow that had been collected from parasitological examination. For this research, they used five deep learning algorithms as a classifier after preprocessing and data augmentation. Moreover, the layers of the applied deep learning models were fine-tuned to optimize the performance and it was found that the model computed accuracy, F1 score, and kappa of 98.7% each. As a result, they proved that by using trained deep learning models with microscopic slide imaging of bone marrow biological material, professionals could precisely detect VL in patients. Gonçalves et al.^14^ detected amastigotes using deep learning techniques in microscopy images. Their proposed method initially segmented the Leishmania parasites in the images and pointed to the position of amastigotes. Their model computed 99.1% accuracy, 80.4% dice, 81.5% precision, 99.6% specificity, 72.2% sensitivity, and 75.2% IoU to identify VL parasites. The researchers mentioned in their paper that their findings were great and demonstrated that deep learning models can be useful to assist specialists in detecting VL in humans after being trained with microscopic images. Rajasekar et al.^15^ mentioned the usage of artificial intelligence for automating the identification of parasite eggs in the laboratory. They applied Detectron2, YOLOs, InceptionV3, and YOLOv8 models to detect parasite eggs and their results showcased that YOLOv8 on incorporating with SGD optimizer did well by computing the mean precision of 0.92 and 98% of F1 score. Based on these results, the researchers said that it was an outstanding model that could be used for the identification of parasite eggs. Masud et al.^16^ examined the application of deep learning algorithms for the identification of malaria via mobile healthcare solutions. A convolutional neural network model was developed along with a cyclical stochastic gradient descent optimizer with an automatic rate finder for examining its performance. The researchers claimed that their proposed model very well classified infected and healthy cells with great precision and accuracy of 97.30%. In fact, their paper's findings could help with the shift of malaria microscopy diagnosis to a mobile application, improving treatment reliability and addressing a shortage of medical knowledge in some areas.

## Methodology

**Figure 1:**
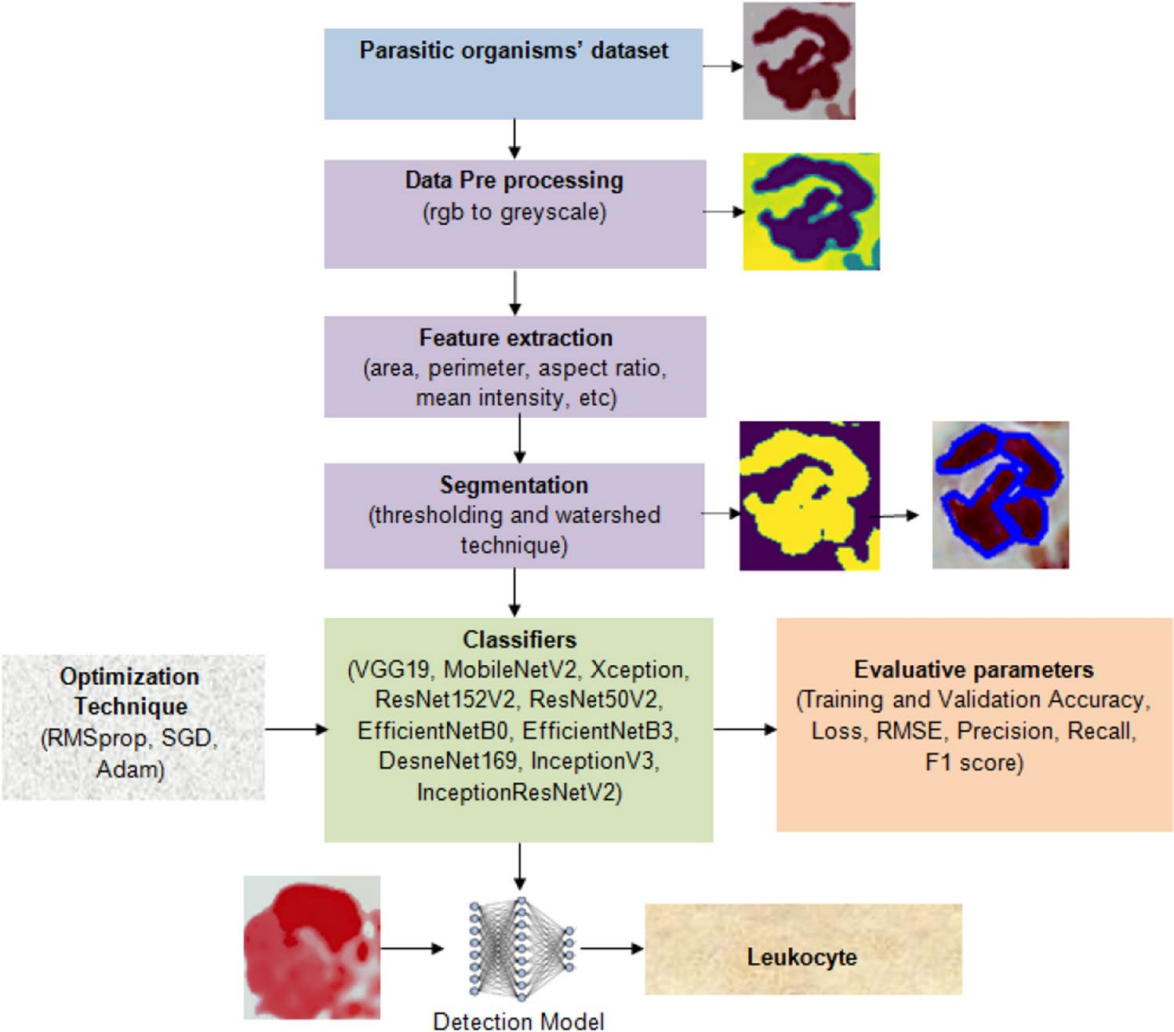
Proposed system to detect and classify parasitic organisms using deep transfer learning.

This section describes the process that has been used to develop the deep learning-based system for the detection and classification of parasitic organisms, as shown in Fig. 1. The proposed system has several notable advantages when it comes to classifying parasitic organisms. The system is able to achieve higher accuracy in detection and classification by using a diverse dataset and advanced image processing techniques. Adding host cells like red and a white blood cell makes the dataset more realistic and relevant to real-life situations. Moreover, when we extract morphological features, we gain a more detailed understanding of the characteristics of the image. The Otsu thresholding and watershed techniques are used to accurately identify regions of interest, which helps to refine the system's focus. By using a range of advanced deep transfer learning models, we can improve its adaptability and overall performance. Moreover, the flexibility of the model is enhanced by fine-tuning its parameters using various optimizers. After carefully evaluating and comparing different factors, we can confidently say that the system is very effective for classifying parasitic organisms in medical imaging datasets.

### Dataset description

**Figure 2:**
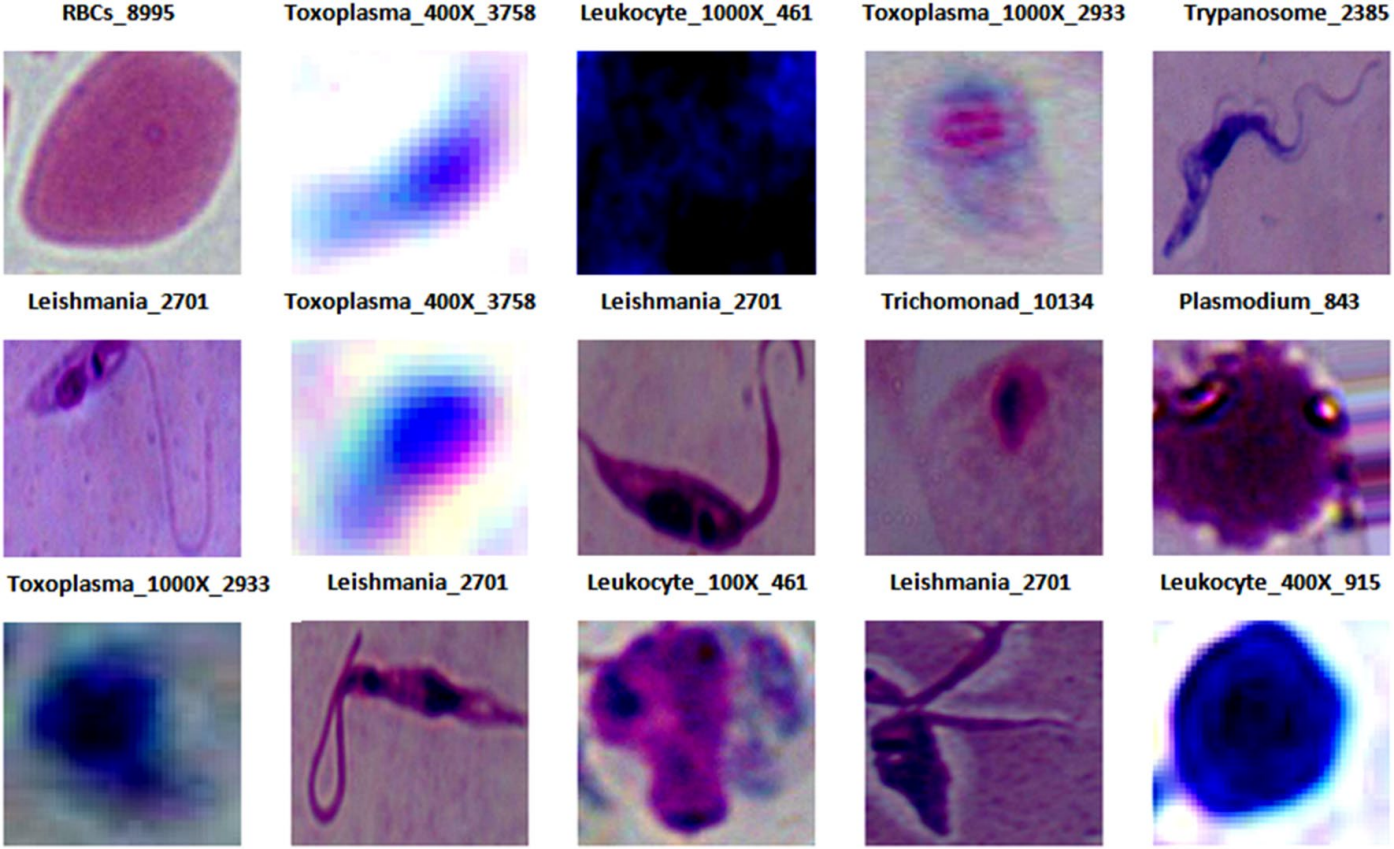
Image samples of parasites.

In this study, a comprehensive data set comprising a total of 34,298 observations was gathered which include various parasites such as Plasmodium, Leishmania, Babesia, Toxoplasma Gondii (T.gondii), Trichomonad, and Trypanosome. Also, the data set includes host cells i.e. Red blood cells and Leukocytes, as shown in Fig. 2. All these images were created using either a 400× or 1000× microscope^17^.

**Figure 3:**
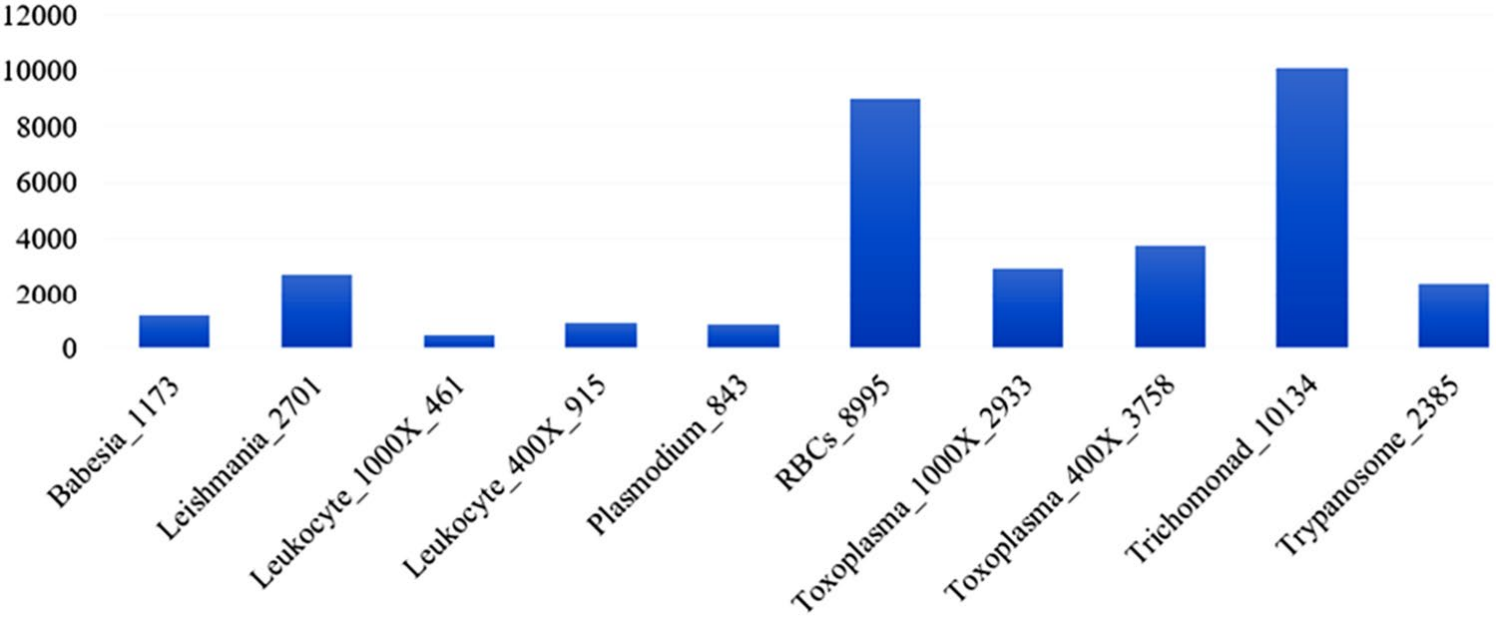
Number of images per class.

Specifically, it comprises 843 instances of Plasmodium, 3758 instances of T.gondii observed under a 400× microscope, and 2933 instances of T.gondii observed under a 1000× microscope. Additionally, the dataset contains 1173 instances of Babesia, 2701 instances of Leishmania, 2385 instances of Trypanosome, and 10,134 instances of Trichomonad, all of which were observed under a 1000× microscope. In addition, the aforementioned dataset comprises a total of 8995 red blood cells (RBCs) and 461 leukocytes observed under a magnification of 1000×, as shown in Fig. 3. Furthermore, an additional 915 leukocytes were identified using a 400× microscope.

### Data preprocessing

**Figure 4:**
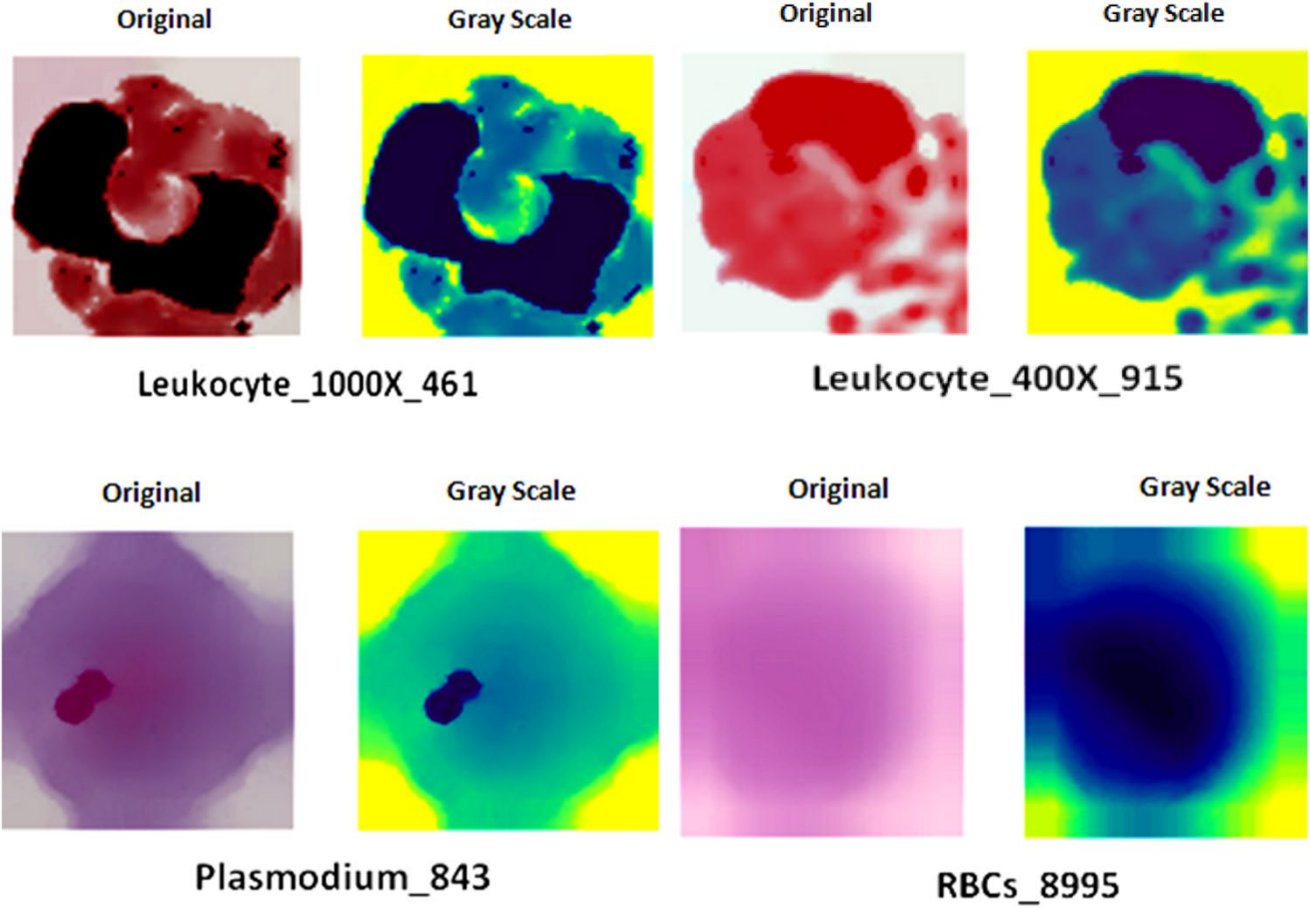
Preprocessing of original images.

Preprocessing of images is a crucial step in image processing as it enables to improves the classification of a model. The first part of the methodology involves loading the original color image, which is represented in the RGB format, using the OpenCV library. Apart from this, the Python Imaging Library (PIL) is also used to handle numerous preprocessing tasks related to images, such as opening, processing, and saving. Later, the image was converted to a single-channel grayscale image whose values range from 0 to 255 using cv2.cvtColor () which specifies cv2.COLOR_RGC2GRAY, as presented in Fig. 4. The purpose of doing so is to reduce the computational complexity of the image data and improve the processing speed. Moreover, greyscale images retain essential features like edges and textures, making them suitable for tasks like object detection, image classification, and feature extraction.

### Feature extraction

As we are aware that the dataset only contains images, we computed the morphological values as shown in Table 1a and b of all the features in the images using parameters such as area, diameter, aspect ratio, minimum and maximum location, etc. for extracting the feature. These values are computed by using the Eqs. (1) to (16)

**Table 1:** Morphological values of the images.

(a) Parameter	Babesia_1173	Leishmania_ 2701	Leukocyte_ 1000X_461	Leukocyte_ 400X_915	Plasmodium_ 843
Area	5.5	0.0	21.0	5.5	2.0
Perimeter	9.07	4.82	29.79	15.414	5.65
Epsilon	0.907	0.482	2.97	1.5414	0.565
Width	4	3	12	2	3
Height	4	2	9	8	3
Aspect ratio	1.0	1.5	1.33	0.25	1.0
Extent	0.34	0.0	0.19	0.343	0.22
Diameter	2.64	0.0	5.17	2.646	1.59
Min value	126.0	129.0	128.0	133.0	127.0
Max value	129.0	130.0	160.0	182.0	133.0
Min value loc	87,196	296,94	36,76	75,78	87,168
Max value loc	87,196	295,93	41,80	75,82	88,168
Mean color	127.80	129.66	137.75	157.92	129.4
Extreme leftmost	85,196	295,93	35,76	74,80	86,168
Extreme rightmost	88,196	297,94	46,82	75,78	88,168
Extreme topmost	87,194	295,93	36,75	75,78	87,167
Extreme bottommost	87,197	296,94	44,83	74,85	87,169

(b) Parameter	RBCs_8995	Toxoplasma_ 1000X_2933	Toxoplasma_ 400X_3758	Trichomonad_ 10,134	Trypanosome_ 2385
Area	4.0	74.0	66.5	0.0	7.0
Perimeter	7.65	34.62	85.21	0.0	9.65
Epsilon	0.76	3.46	8.521	0.0	0.96
Width	4	12	16	1	4
Height	3	12	11	1	4
Aspect ratio	1.33	1.0	1.45	1.0	1.0
Extent	0.33	0.513	0.377	0.0	0.4375
Diameter	2.25	9.706	9.201	0.0	2.98
Min value	127.0	34.0	128.0	128.0	126.0
Max value	133.0	155.0	228.0	128.0	129.0
Min value Loc	88,184	10,10	7,1	63,135	199,262
Max value Loc	87,184	5,11	14,0	63,135	198,261
Mean color	129.375	107.95	168.33	128.0	128.08
Extreme leftmost	87,184	5,14	0,0	63,135	197,262
Extreme rightmost	90,184	16,7	15,10	63,135	200,262
Extreme topmost	88,183	10,5	0,0	63,135	198,261
Extreme bottommost	89,185	9,16	0,10	63,135	199,264

1 $$area=height*width$$

2 $$height=cv2.boundingRect\left(cnt\right)$$

3 $$width=cv2.boundingRect\left(cnt\right)$$

4 $$Aspect \; Ratio= \frac{width}{height}$$

5 $$Extent= \frac{object \;area}{bounding \;rectangle \,area}$$

6 $$Equivalent\; diameter= \sqrt{\frac{4*contour \;area}{\pi }}$$

7 $$epsilon= \sqrt{{(({x}_{2}-{x}_{1})}^{2}+{({y}_{2}-{y}_{1} )}^{2}}$$

8 $$Minimum \;value=cv2.{\text{min}}()$$

9 $$Maximum \;value=cv2.{\text{max}}()$$

10 $$Minimum \;value \; Location=cv2.{\text{minMaxLo}}()$$

11 $$Maximum \;value \; Location=cv2.{\text{minMaxLo}}()$$

12 $$Mean\; Color=cv2.{\text{mean}}()$$

13 $$Extreme\; Leftmost\; point=tuple(cnt(cnt\left[:,:,0\right].argmin()\left[0\right])$$

14 $$Extreme \;Rightmost \;point=tuple(cnt(cnt\left[:,:,0\right].argmin()\left[0\right])$$

15 $$Extreme \;Topmost \;point=tuple(cnt(cnt\left[:,:,1\right].argmin()\left[0\right])$$

16 $$Extreme \;Bottommost \;point=tuple(cnt(cnt\left[:,:,1\right].argmin()\left[0\right])$$

### Data segmentation

**Figure 5:**
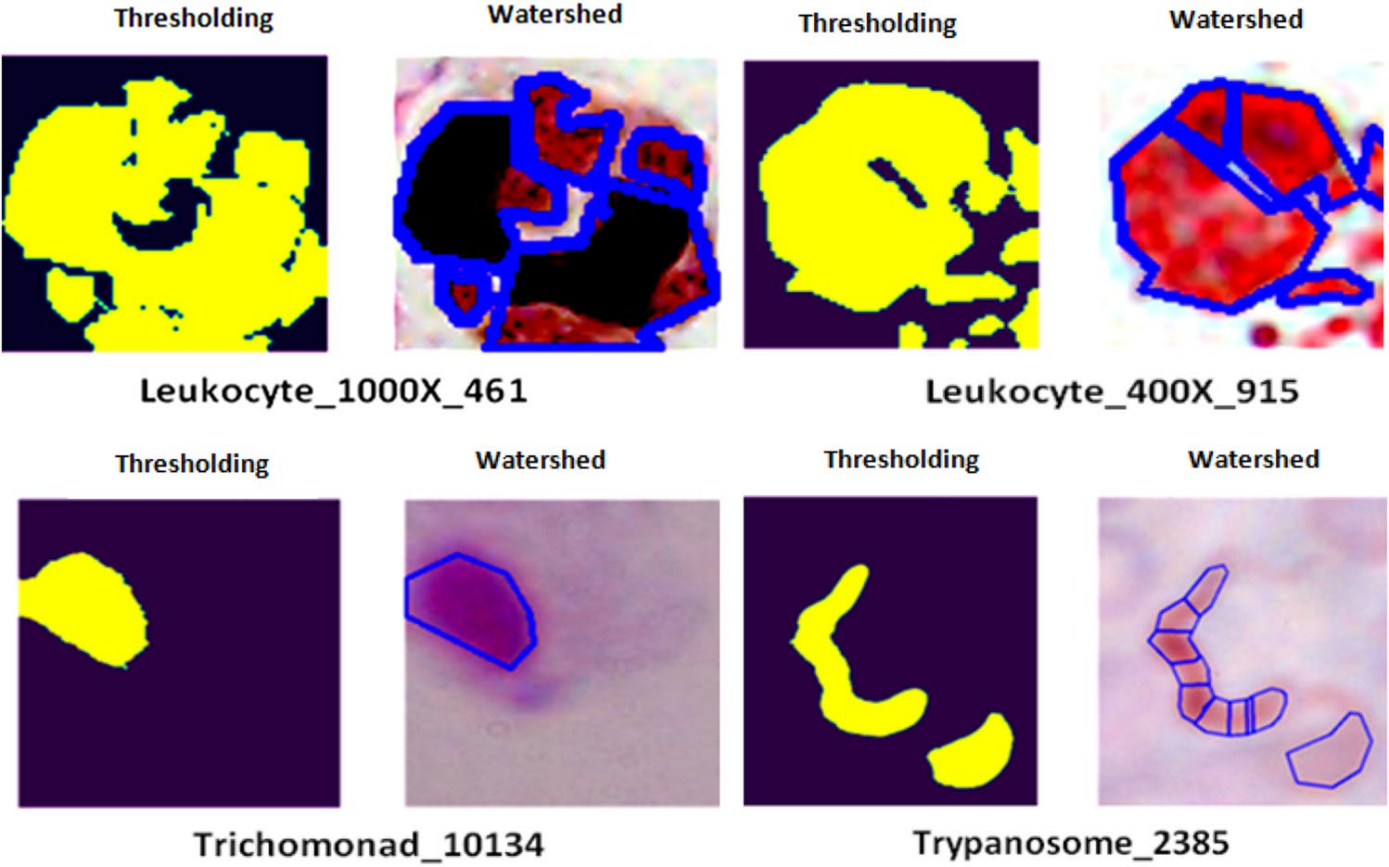
Segmentation of images.

Image segmentation is an important task and incorporating thresholding with the watershed technique is the most used technique to segment objects or regions of interest within an image, as shown in Fig. 5. Initially, thresholding of image has been done using the Otsu thresholding technique which automatically determines an optimal threshold for segmenting the images by minimizing the inter-class variance^18^. Mathematically it is represented by the Eqs. (17, 18):

Let’s $P(i)$ be the probability of a pixel with intensity $i$ in the image and to calculate total probability ${P}_{t}$

17 $${P}_{t}= \sum_{i=0}^{L-1}P(i),$$

where L is the number of possible intensity levels. The mean intensity (${\mu }_{t})$ of the pixels in the foreground is given by

18 $${\mu }_{t}= \sum_{i=0}^{L-1}i .P(i)$$

And to calculate the between class variance ${\sigma }_{B}^{2}(t)$, the equation (xix) is:

19 $${\sigma }_{B}^{2}\left(t\right)= P\left(t\right).\left(1-P\left(t\right)\right).{({\mu }_{total }.P\left(t\right)-{\mu }_{t})}^{2}$$

where ${\mu }_{total}$ is the mean intensity of the entire image and ${\mu }_{total }= \sum_{i=0}^{L-1}i .P(i)$. Additionally, the aim of Otsu method is to maximize ${\sigma }_{B}^{2}\left(t\right)$ by finding the threshold *t* that satisfies (equation xx):

20 $${t}_{Otsu}={argmax}_{t}{\sigma }_{B}^{2}\left(t\right)$$

The optimal threshold ${t}_{Otsu}$ obtained from this maximization process is then used for binary thresholding to separate the image into foreground and background based on pixel intensities.

As thresholding would not have been sufficient hence to refine it watershed technique has been applied where the image is treated as topographic map. The watershed algorithm can be mathematically represented using the gradient of the image $(\nabla f)$. The gradient magnitude of an image is calculated using derivatives in the x and y directions ($\frac{\partial f}{\partial x} and \frac{\partial f}{\partial y})$. The watershed transformation is usually defined in Eq. (21):

21 $$Watershed \left(f\right)=\left\{x \in Image \right| \nabla f(x)=0 \; and \; x \; is \; a \; local\; minimum\}$$

Here, $\nabla f(x)=0$ corresponds to the points where the gradient is zero, indicating flat regions in the image. These points are the markers for the watershed segmentation process. Watershed segmentation considers these markers and the gradient of the image to delineate the regions accurately.

Such a combination of Otsu thresholding followed by the watershed technique is a versatile method for segmenting the images. Otsu Thresholding provides an initial separation between object and background, while the watershed step refines the boundaries based on local image characteristics.

### Applied models

The usage of advanced deep learning models, whose layers are either fine-tuned or employed as feature extractors, enhances the prediction capabilities in visual recognition tasks. Likewise, in the realm of parasitic organism detection and classification, various specialized neural network architectures have been developed and briefly explained^19^.

The use of **VGG19**, a convolutional neural network architecture well-known for its depth as well as hierarchical feature learning capabilities, has proven to be of huge value in the task of discriminating complicated patterns within parasitic images. By leveraging its abundant layers, VGG19 enables the accurate classification of these parasites, thereby enhancing the precision of identification^20^. Applying the various filter sizes in **Inception V3** allows for the comprehensive capture of a broad range of features, which is essential in the precise detection of various parasitic organisms^21^. The architecture of **EfficientNetB3** is designed to achieve stability between accuracy as well as computational efficiency. This makes it well-suited to analyze large and diverse datasets that are usually encountered in the field of analyzing parasitic organisms^22^.

The **ResNet152V2** and **ResNet50V2** architectures have been designed to deal with the vanishing gradient problem which is generally encountered during training deep neural networks. These models include skip connections and residual blocks to deal with mitigating the issue. By applying these architectural components, the models can maintain stable training even when dealing with complex parasitic images^23,24^. **MobileNetV**2 is a convolutional neural network architecture that aims to reduce computational costs to maintain high accuracy. This makes it particularly suitable for environments with limited resources^25^. The dense connections in **DenseNet169** enable a comprehensive analysis of parasitic images, allowing for a detailed examination of various aspects^26^. On the other hand, **EfficientNetB0's** systematic scaling strategy guarantees superior performance while minimizing computational requirements. This characteristic is particularly advantageous in real-time applications for detecting parasitic diseases, where efficiency and accuracy are crucial factors^27^.

On the other hand, **Xception** is another convolutional neural network architecture that incorporates unique convolutional layers and skip connections. These architectural choices are designed to improve the flow of gradients during training, which in turn helps in the detection of subtle parasitic features^28^. **InceptionResNetV2,** a novel deep neural network architecture, combines the advantageous characteristics of Inception and ResNet models. By incorporating Inception's multiscalar feature capturing capability and ResNet's depth, ResNetV2 demonstrates exceptional performance in intricate parasitic image recognition tasks, such as object detection and segmentation. The detection and classification of parasitic diseases present a range of challenges that are addressed by various architectural approaches. Each architecture offers distinct advantages that collectively contribute to the overall effectiveness of the detection and classification process^29^. The general layered architecture of all these applied models has been shown in Table 2.

**Table 2:** Layered architecture of applied deep learning models.

Models	Convolutional layer	Pooling layer	Dense Layer	Other architectural features	Parameters
VGG19	16	5	3	None	143 M
Inception V3	48	8	3	Batch normalization	23 M
EfficientNetB3	9	7	2	Squeeze-and-excitation	6.8 M
ResNet152V2	152	39	3	Residual connections	60.2 M
ResNet50V2	50	13	3	Residual connections	25.6 M
MobileNetV2	18	6	2	Inverted residual connections	3.5 M
Xception	36	11	3	Depthwise separable convolutions	22.9 M
Densenet169	169	5	3	Dense connections	14.3 M
EfficientNetB0	5	4	2	Squeeze-and-excitation	5.3 M
InceptionResNetV2	164	41	3	Batch normalization	55 M

There are also some additional details about the rest of the architectural features which have been mentioned in the aforementioned table:

- *Batch normalization:* This technique is useful for the normalization of inputs for each layer of a neural network in order to improvise the performance and stability of the network.
- *Squeeze and excitation:* This technique compresses and excites the feature maps which has been produced by each layer and thereby improves the efficiency of neural network
- *Residual connections:* This also helps to improve the performance of the network by working on the vanishing gradient issue and is also used as shortcut layers of a neural network.
- *Inverted residual connections:* These connections more efficient and better than the traditional residual connections.
- *Dense connections:* These techniques are used for connecting each layer of a neural network with all its preceding layers so that each of these layers can learn from the previous layers and improve the performance of the network.

### Performance metrics

In the context of deep learning models, there are various metrics which can be used to examine their performance which are described as following^20–36^:

#### Accuracy

It is a very fundamental metric which is the ratio of correctly predicted classes of total classes in the dataset (Eq. 22). It is used to inform us how well the model is performing for the particular dataset but on the contrary, this metric does not work well when the data is imbalanced.

22 $$Accuracy= \frac{True \;Positive+True\; Negative}{True \;Positive+True \;Negative+False \;Positive+False \;Negative}$$

#### Loss

Like accuracy, loss is also an important metric to examine the performance of the model. It works exactly the opposite to as it computes the error by generating the difference between the predicted and actual target values (Eq. 23). If the value of loss is high that means the model has not been trained well and if it is low, it means the model has been trained well and will predict the output correctly.

23 $$Loss= \frac{{(Actual \;Value-Predicted \;Value)}^{2}}{Number \;of \;observations}$$

#### Root mean square error (RMSE)

This metric is also calculated by taking the difference of predicted and actual value but it quantifies the average magnitude of the value or error generated (Eq. 24).

24 $$RMSE= \sqrt{\frac{{(Actual\; Value-Predicted \;Value)}^{2}}{Number\; of \;observations}}$$

#### Precision and recall

These two metrics work particularly in those scenarios where the dataset is imbalanced. Precision measures the accuracy of the classes that have been predicted positively and is calculated as the ratio of true positive prediction to the total positive prediction (Eq. 25). On the other hand, recall measures the ability of the model to identify all the relevant classes and is calculated as the ratio of true positives to actual positives (Eq. 26).

25 $$Precision= \frac{True\; Positive}{True \;Positive+False \;Positive}$$

26 $$Recall= \frac{True \;Positive}{True \;Positive+False \;Negative}$$

#### F1 score

It is a combination or a harmonic mean of precision as well as recall which considers both false negative and false positive as well as provides a balanced assessment of any model’s performance (Eq. 27).

27 $$F1 score=2\frac{Precision*Recall}{Recall+Precision}$$

## Results

In this section, we conduct a full analysis of the models based on a set of parameters outlined in "Performance metrics" section. We have evaluated the performance of the models after applying three different optimizer techniques which include RMSprop, SGD, and Adam optimizer. These optimizers optimize the parameters or variables in the form of weights and biases of the layers of deep transfer learning techniques which determine the mapping between input features and output predictions^37^. Our assessment encompasses both the training and validation datasets, allowing us to compare the models for whole and various classes of datasets.

### RMSprop

Initially, the applied deep learning models are optimized by using RMSprop optimizer and are examined during both training and validation phases, as shown in Table 3.

**Table 3:** Evaluation of models during training and validation phases after applying RMSprop.

Models	Training records			Validation records		
	Accuracy	Loss	RMSE value	Accuracy	Loss	RMSE value
VGG19	99.89	0.11	0.33	99.91	0.09	0.31
Inception V3	99.83	0.12	0.34	99.88	0.10	0.31
EfficientNet B3	99.70	0.13	0.36	99.91	0.12	0.34
ResNet152 V2	99.64	0.14	0.37	99.41	0.12	0.34
ResNet50 V2	99.63	0.13	0.36	98.13	0.16	0.40
MobileNet V2	99.26	0.23	0.47	98.95	0.19	0.43
Xception	99.17	0.31	0.55	96.26	0.29	0.53
DenseNet 169	99.61	0.13	0.36	99.50	0.11	0.33
EfficientNet B0	99.49	0.14	0.37	99.91	0.09	0.30
InceptionResNetV2	99.99	0.12	0.34	99.44	0.11	0.33

On analyzing the models, it can be seen that all models did quite well but during the training period, it has been found that InceptionResNetV2, VGG19, and InceptionV3 performed well by computing 99.99%, 99.89%, and 99.83% accuracies respectively along with the lowest loss and RMSE values as compared to the others. On the contrary, it has been found that VGG19, InceptionV3, and EfficientNetB0 worked well for the validation dataset by obtaining an accuracy of 99.91% each. As far as loss and RMSE values are concerned, only VGG19 and EfficientNetB0 stood at the best place with 0.09 each.

**Figure 6:**
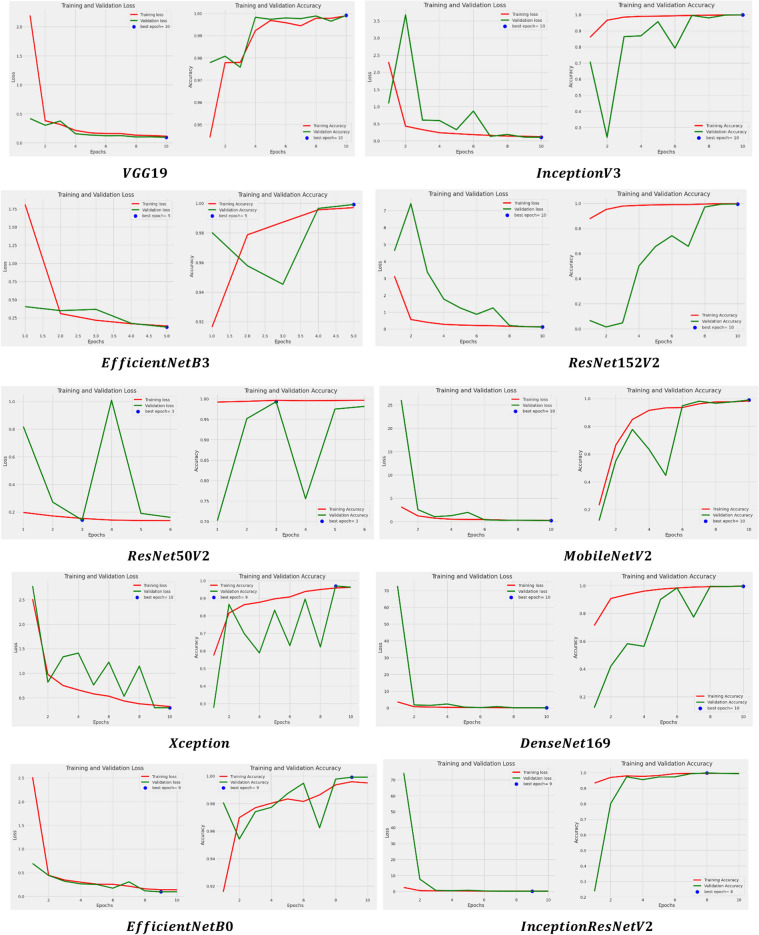
Learning curves of applied models after applying RMSprop.

In addition to this, the models are examined based on their learning curves of accuracy and loss during both training and validation periods as shown in Fig. 6.

All models underwent a rigorous 10-epoch iteration process, during which they consistently demonstrated their optimal performance either at the 10th epoch or between the 8th and 10th epochs. This observation holds true for both accuracy and loss metrics. However, it's noteworthy that certain models exhibited significant disparities in their performance. These disparities raise a red flag, suggesting a potential issue with overfitting in those models. Overfitting occurs when a model learns the training data too well, including its noise and outliers, leading to poor generalization on unseen or validation data. Identifying and addressing this overfitting concern is crucial for enhancing the overall robustness and reliability of the models. Other than this, the models are also evaluated for another set of parameters i.e. precision, recall, and F1 score whose results are shown in Table 4.

**Table 4:** Analysis of models for different parameters after applying RMSprop.

Models	Precision	Recall	F1 score
VGG19	0.99	1.00	1.00
Inception V3	0.99	1.00	1.00
EfficientNet B3	0.99	0.99	0.99
ResNet152 V2	0.99	0.99	0.99
ResNet50 V2	0.98	1.00	0.99
MobileNet V2	0.98	0.99	0.98
Xception	0.94	0.96	0.95
DenseNet 169	0.99	1.00	0.99
EfficientNet B0	0.99	1.00	0.99
InceptionResNetV2	0.99	0.99	0.99

VGG19 and Inception V3 have high precision, recall, and F1 scores, indicating that they perform very well in the classification task, achieving near-perfect accuracy and completeness. EfficientNet B3, ResNet152 V2, DenseNet 169, and EfficientNet B0 also have high precision and F1 scores but slightly lower F1 score compared to VGG19 and Inception V3. ResNet50 V2 and MobileNet V2 have slightly lower precision compared to the top-performing models but still achieve high recall and F1 scores. Xception has the lowest precision, recall, and F1 score among the listed models, indicating that it may have more false positives and false negatives compared to the other models.

**Figure 7:**
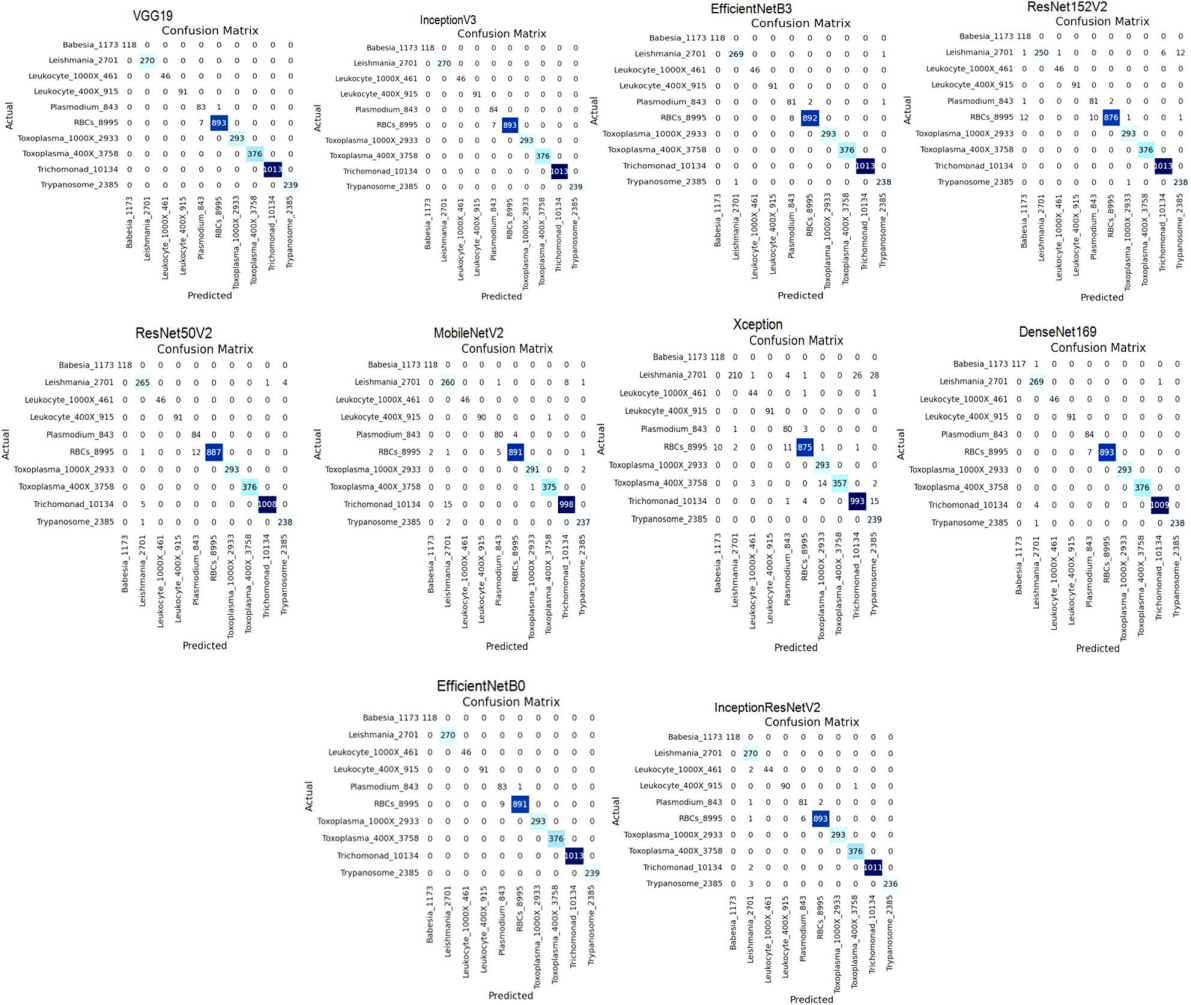
Confusion matrix of applied models after applying RMSprop.

After examining the performance of the models for the classification of various parasitic organisms, confusion matrices of 10 × 10 have been generated which is a crucial tool for assessing the performance of multi-class classification models, as shown in Fig. 7. Based on these matrices, true positive, true negative, false positive, and false negative values are being taken to examine the performance of models for different classes, as shown in Table 5.

**Table 5:** Evaluation of models during training and validation phases for different classes after applying RMSprop.

Classes	Models	Training records			Validation records		
		Accuracy	Loss	RMSE	Accuracy	Loss	RMSE
Babesia_1173	VGG19	98.92	0.20	0.44	99.43	0.24	0.48
	Inception V3	98.85	0.28	0.52	99.19	0.26	0.50
	EfficientNet B3	98.98	0.49	0.70	99.49	0.25	0.50
	ResNet152 V2	99.49	0.29	0.53	99.46	0.28	0.52
	ResNet50 V2	99.46	0.49	0.70	99.49	0.32	0.56
	MobileNet V2	99.9	0.59	0.76	99.59	0.31	0.55
	Xception	98.49	0.34	0.58	99.29	0.36	0.60
	DenseNet 169	99.46	0.24	0.48	99.19	0.26	0.51
	EfficientNet B0	99.46	0.29	0.53	99.19	0.34	0.58
	InceptionResNetV2	99.89	0.15	0.38	99.76	0.23	0.47
Leishmania_2701	VGG19	91.31	4.9	2.21	99.05	0.28	0.52
	Inception V3	95.82	1.79	1.33	99.36	0.086	0.29
	EfficientNet B3	98.23	0.359	0.59	98.38	0.316	0.56
	ResNet152 V2	96.36	1.022	1.01	64.63	149.33	12.22
	ResNet50 V2	54.21	343.04	18.52	95.63	3.09	1.75
	MobileNet V2	88.67	2.36	1.53	99.63	0.89	0.94
	Xception	98.59	0.49	0.70	99.67	0.138	0.37
	DenseNet 169	96.36	2.89	1.70	99.05	0.28	0.52
	EfficientNet B0	99.59	2.16	1.49	99.88	1.14	0.89
	InceptionResNetV2	99.99	1.12	1.59	99.96	0.53	0.49
Leukocyte_400X_915	VGG19	98.59	0.359	0.59	99.85	0.14	0.56
	Inception V3	99.56	0.246	0.49	99	0.16	0.59
	EfficientNet B3	99.76	0.049	0.22	98.4	0.39	0.62
	ResNet152 V2	98.49	0.376	0.61	99.29	0.28	0.52
	ResNet50 V2	54.21	343.04	18.52	98.46	0.02	0.14
	MobileNet V2	88.67	2.36	1.53	97.31	0.34	0.59
	Xception	98.59	0.49	0.70	62.63	159.46	12.62
	DenseNet 169	96.36	2.89	1.70	94.59	3.50	1.87
	EfficientNet B0	98.59	0.359	0.59	96.49	0.89	0.94
	InceptionResNetV2	99.99	0.12	1.29	99.94	0.15	0.39
RBCs_8995	VGG19	99.92	0.16	0.85	99.85	0.14	0.59
	Inception V3	99.85	0.18	0.59	99.49	0.16	0.67
	EfficientNet B3	98.59	0.35	0.59	98.59	0.35	0.59
	ResNet152 V2	97.56	0.24	0.49	99.56	0.24	0.49
	ResNet50 V2	98.76	0.05	0.24	99.76	0.04	0.22
	MobileNet V2	96.49	0.34	0.58	98.49	0.37	0.61
	Xception	62.29	149.59	12.23	64.29	149.35	12.22
	DenseNet 169	94.46	3.04	1.74	95.46	3.04	1.74
	EfficientNet B0	98.76	0.84	0.92	99.76	0.85	0.92
	InceptionResNetV2	99.49	0.14	0.38	99.94	0.17	0.41
Toxoplasma_400X_3758	VGG19	99.92	0.16	0.46	91.49	4.949	2.22
	Inception V3	97.45	0.349	0.59	95.46	1.776	1.33
	EfficientNet B3	94.05	0.276	0.52	98.19	0.316	0.56
	ResNet152 V2	99.36	0.046	0.21	96.46	1	1
	ResNet50 V2	94.38	0.319	0.56	54.76	300.94	17.34
	MobileNet V2	66.63	149.73	12.23	88.59	2.29	1.51
	Xception	98.63	3.049	1.74	98.22	0.489	0.69
	DenseNet 169	97.63	0.876	0.93	96.76	2.846	1.68
	EfficientNet B0	96.67	0.129	0.35	91.49	4.949	2.22
	InceptionResNetV2	99.99	0.12	0.26	99.97	0.18	0.24
Trypanosome_2385	VGG19	99.92	0.16	0.59	98.52	0.349	0.59
	Inception V3	90.31	3.9	1.97	99.29	0.76	0.87
	EfficientNet B3	94.49	2.79	1.67	98.76	0.492	0.70
	ResNet152 V2	97.26	0.359	0.59	97.19	0.355	0.59
	ResNet50 V2	98.59	4.022	2.01	62.46	159.4	12.62
	MobileNet V2	56.21	43.04	6.56	94.49	3	1.73
	Xception	87.67	2.59	1.61	96.55	0.89	0.94
	DenseNet 169	96.59	0.86	0.92	99.29	0.109	0.33
	EfficientNet B0	98.36	2.59	1.61	98.52	0.349	0.59
	InceptionResNetV2	90.31	3.9	1.97	99.90	0.33	0.65
Leukocyte_1000X_461	VGG19	98.52	0.34	0.59	99.3	0.44	0.66
	Inception V3	99.29	0.76	0.87	99.15	0.88	0.93
	EfficientNet B3	98.76	0.49	0.70	97.72	0.35	0.59
	ResNet152 V2	97.19	0.35	0.59	97.75	1.42	1.19
	ResNet50 V2	62.46	159.4	12.62	97.3	0.53	0.72
	MobileNet V2	94.49	3	1.73	94.63	1.39	1.17
	Xception	96.55	0.89	0.94	98.72	1.27	1.12
	DenseNet 169	99.29	0.11	0.33	99.15	1.55	1.24
	EfficientNet B0	98.52	0.34	0.59	99.3	0.44	0.66
	InceptionResNetV2	99.29	0.76	0.87	99.32	0.88	0.93
Plasmodium_843	VGG19	99.92	0.36	0.66	99.85	0.44	0.89
	Inception V3	99.92	0.16	0.46	91.49	4.94	2.22
	EfficientNet B3	97.45	0.34	0.59	95.46	1.77	1.33
	ResNet152 V2	94.05	0.27	0.52	98.19	0.31	0.56
	ResNet50 V2	99.36	0.04	0.21	96.46	1	1
	MobileNet V2	94.38	0.31	0.56	54.76	300.94	17.34
	Xception	66.63	149.73	12.23	88.59	2.29	1.51
	DenseNet 169	98.63	3.04	1.74	98.22	0.48	0.69
	EfficientNet B0	99.92	0.16	0.46	91.49	4.94	2.22
	InceptionResNetV2	99.99	0.12	0.26	99.96	0.13	0.26
Toxoplasma_1000X_2933	VGG19	99.92	0.16	0.85	99.85	0.14	0.59
	Inception V3	99.85	0.18	0.59	99.94	0.16	0.67
	EfficientNet B3	98.59	0.35	0.59	98.59	0.35	0.59
	ResNet152 V2	97.56	0.24	0.49	99.56	0.24	0.49
	ResNet50 V2	98.76	0.05	0.24	99.76	0.04	0.22
	MobileNet V2	96.49	0.34	0.58	98.49	0.37	0.61
	Xception	62.29	149.59	12.23	64.29	149.35	12.22
	DenseNet 169	94.46	3.04	1.74	95.46	3.04	1.74
	EfficientNet B0	98.76	0.84	0.92	99.76	0.85	0.92
	(InceptionResNetV2)	99.49	0.14	0.38	99.49	0.17	0.41
Trichomonad_10134	VGG19	98.92	0.20	0.44	99.43	0.24	0.48
	Inception V3	98.85	0.28	0.52	99.19	0.26	0.51
	EfficientNet B3	98.98	0.49	0.70	99.49	0.25	0.50
	ResNet152 V2	99.49	0.29	0.53	99.46	0.28	0.52
	ResNet50 V2	99.46	0.49	0.70	99.79	0.32	0.56
	MobileNet V2	99.9	0.59	0.76	99.59	0.31	0.55
	Xception	98.49	0.34	0.58	99.29	0.36	0.60
	DenseNet 169	99.46	0.24	0.48	99.19	0.26	0.51
	EfficientNet B0	99.46	0.29	0.53	99.19	0.34	0.58
	InceptionResNetV2	99.89	0.15	0.38	99.46	0.23	0.47

In the comprehensive analysis of various models across multiple datasets, InceptionResNetV2 consistently emerges as the top performer, exhibiting exceptional accuracy and low loss across most datasets, including Babesia_1173, Leishmania_2701, Leukocyte_400X_915, RBCs_8995, Toxoplasma_400X_3758, Leukocyte_1000X_461, and Plasmodium_843. Notably, ResNet50V2 and Xception did not perform well in Trypanosome_2385 and Toxoplasma_1000X_2933 as they computed 62.46% and 64.29% accuracy respectively whereas MobileNetV2 excels in Trichomonad_10134 with 99.59% accuracy. These variations highlight the nuanced performance of different models across diverse datasets. Additionally, the overall dominance of InceptionResNetV2 signifies its robustness and reliability, making it the preferred choice for most classes of datasets, while other models demonstrate specialized efficacy in specific contexts, emphasizing the need for a tailored approach based on the dataset under consideration.

**Figure 8:**
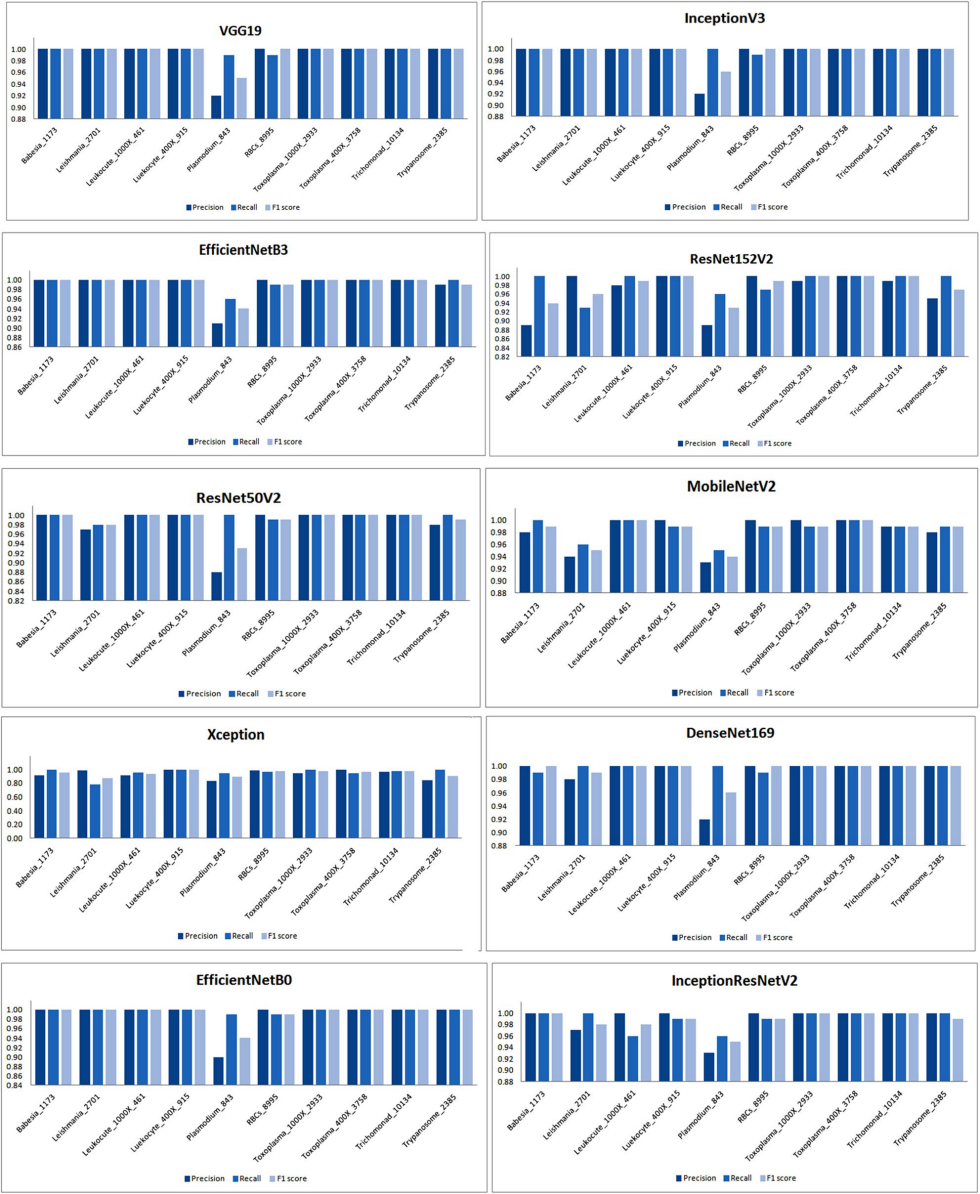
Graphical analysis of models after applying RMSprop optimizer.

Besides this, these models are also examined for other performance metrics for different classes of the dataset whose results are shown graphically in Fig. 8.

### SGD

The applied deep learning models are also optimized by using SGD optimizer and are examined during both training and validation phases, as shown in Table 6.

**Table 6:** Evaluation of models during training and validation phases using SGD optimizer.

Models	Training records			Validation records		
	Accuracy	Loss	RMSE Value	Accuracy	Loss	RMSE value
VGG19	99.27	1.12	1.05	99.85	0.99	0.99
Inception V3	99.81	1.24	1.11	99.91	0.98	0.98
EfficientNet B3	99.65	1.16	1.07	99.73	1.02	1.00
ResNet152 V2	99.65	1.09	1.04	99.24	0.98	0.98
ResNet50 V2	99.72	1.15	1.07	99.62	1.02	1.00
MobileNet V2	99.69	1.16	1.07	99.65	1.02	1.00
Xception	99.47	0.96	0.97	99.59	0.86	0.92
DenseNet 169	99.61	1.17	1.08	99.65	1.03	1.01
EfficientNet B0	99.51	1.14	1.06	99.85	1.00	1
Hybrid (InceptionResNetV2)	99.90	0.90	0.94	99.90	1.00	1

The table shows the performance of 9 different models on a dataset of training and validation records. The metrics used to evaluate the performance of the models are accuracy, loss, and RMSE value. Overall, the models perform very well, with all accuracies above 99%. However, there are some small differences in performance between the models. InceptionResNetV2 has the highest accuracy on both the training dataset with 99.90% accuracy followed by Inception V3 with 99.81% accuracy**.** However, ResNet152 V2, ResNet50 V2, and MobileNet V2 also perform well with training accuracies above 99% but they have slightly lower accuracies on the validation dataset. On the contrary, **Xception** and VGG19 have the lowest accuracy on the training dataset, but they perform well on the validation dataset. Overall, InceptionV3 and InceptionResNetV2 are the best-performing models on this dataset. However, the other models also perform very well but the highest validation accuracy has been computed by InceptionV3 with 99.91% on a loss of 0.98.

**Figure 9:**
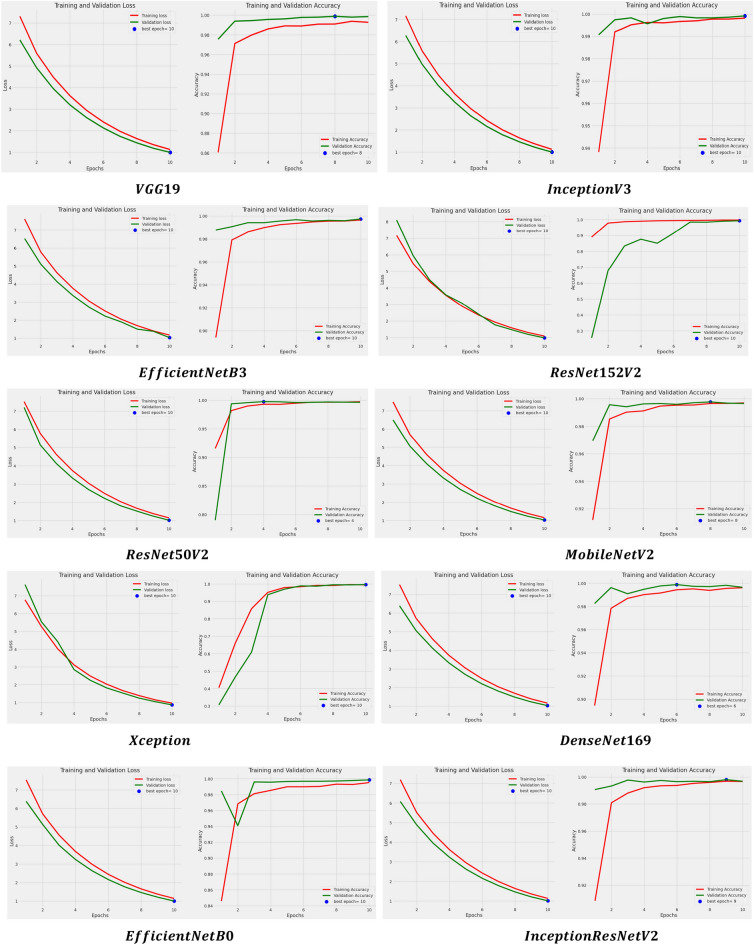
Learning curves of applied models after using SGD optimizer.

In addition to this, the models are examined based on their learning curves of accuracy and loss during both training and validation periods as shown in Fig. 9.

The models went through a thorough process of 10 iterations, where they consistently showed their best performance either at the 10th iteration or between the 8th and 10th iterations. This observation applies to both accuracy and loss metrics. On the contrary, the learning accuracy curve of the models such as InceptionresNetV2, Xception, ResNet50V2, MobileNetV2, EfficientNetB3, ResNet152V2, and InceptionV3 have shown good fit learning curves which means the learning of the model exist between overfit and underfit model. In addition to that, the models are also assessed for another set of parameters: precision, recall, and F1 score. The results for these parameters are displayed in Table 7.

**Table 7:** Analysis of models for different parameters using SGD optimizer.

Models	Precision	Recall	F1 score
VGG19	0.99	1.00	0.99
Inception V3	0.99	1.00	0.99
EfficientNet B3	0.99	1.00	0.99
ResNet152 V2	0.98	0.99	0.99
ResNet50 V2	0.99	1.00	0.99
MobileNet V2	0.99	1.00	0.99
Xception	0.99	1.00	0.99
DenseNet 169	0.99	1.00	0.99
EfficientNet B0	0.99	1.00	1.00
InceptionResNetV2	0.99	0.99	0.99

All of the models in the table perform very well, with precision, recall, and F1 scores above 99%. This indicates that the models are accurately identifying both negative and positive cases in the dataset. InceptionResNetV2 has the highest precision, recall, and F1 scores, followed by Inception V3 and EfficientNet B3. ResNet152 V2, Xception, MobileNet V2, DenseNet 169, ResNet50 V2, and EfficientNet B0 also perform very well, with precision, recall, and F1 scores above 99%. Overall, all of the models perform very well on this dataset.

**Figure 10:**
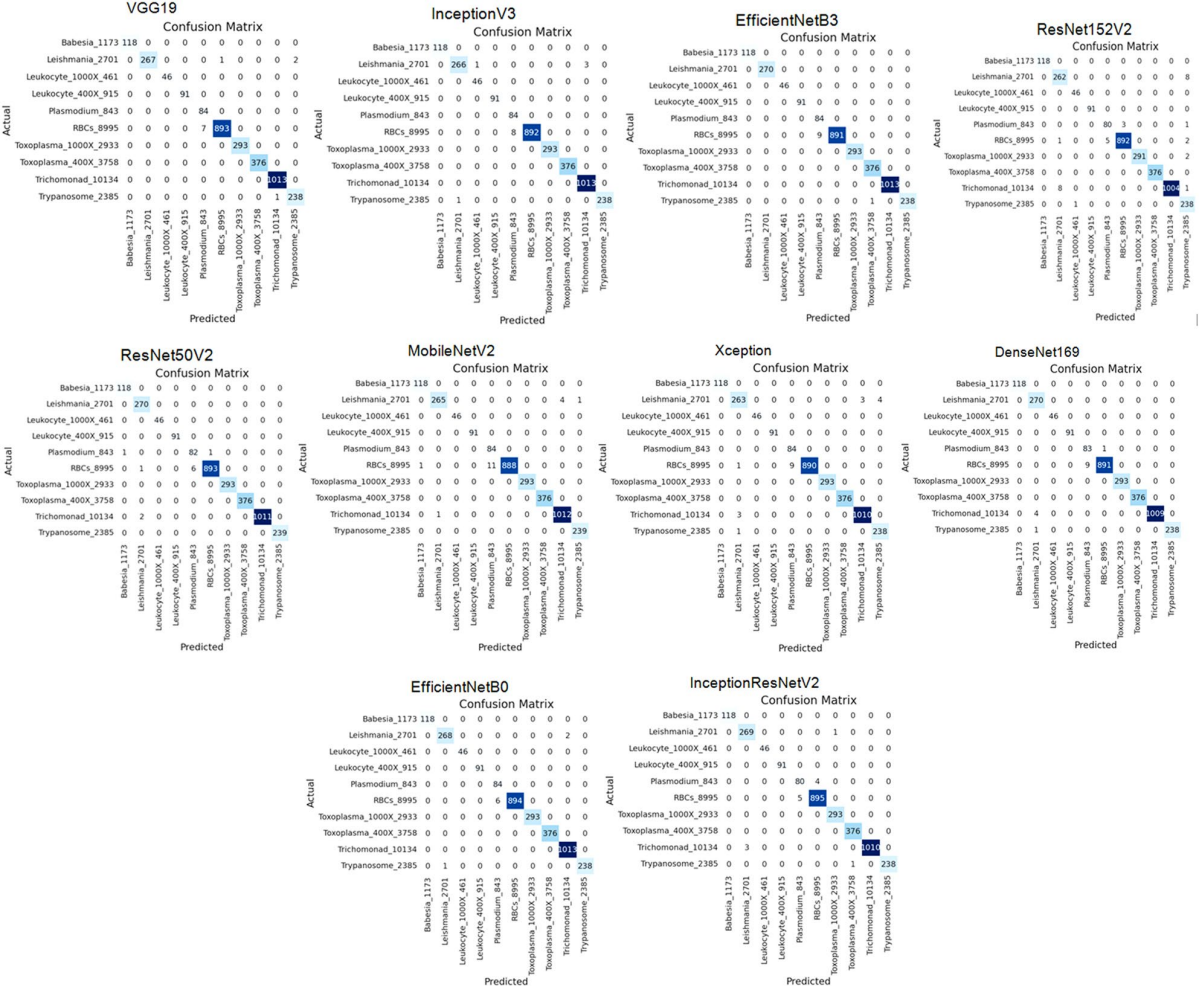
Confusion matrix of applied models of SGD optimizer.

We have generated confusion matrices of size 10 × 10 for evaluating the performance of different models to classify parasitic organisms, as shown in Fig. 10. These matrices are mainly to assess how well the models are performing multi-class classification by using these to examine the performance, as shown in Table 8.

**Table 8:** Evaluation of models during training and validation phases for different classes of SGD optimizer.

Classes	Models	Training records			Validation records		
		Accuracy	Loss	RMSE	Accuracy	Loss	RMSE
Babesia_1173	VGG19	95.84	0.59	0.77	95.84	0.59	0.77
	Inception V3	94.76	0.57	0.76	94.76	0.57	0.76
	EfficientNet B3	92.14	0.46	0.68	98.59	0.08	0.29
	ResNet152 V2	91.33	0.49	0.70	93.75	0.45	0.67
	ResNet50 V2	96.35	0.46	0.68	98.56	0.08	0.29
	MobileNet V2	94.28	0.59	0.76	98.56	0.75	0.87
	Xception	99.86	0.49	0.70	97.86	0.85	0.92
	DenseNet 169	91.61	0.56	0.75	94.26	0.09	0.30
	EfficientNet B0	92.20	0.49	0.70	95.5	0.59	0.77
	InceptionResNetV2	96.84	0.26	0.51	98.78	0.57	0.76
Leishmania_2701	VGG19	98.59	0.03	0.18	75.28	1.30	1.14
	Inception V3	95.26	0.06	0.25	59.40	2.14	1.46
	EfficientNet B3	99.59	0.02	0.16	98.88	0.05	0.22
	ResNet152 V2	94.59	0.05	0.23	86.32	0.12	0.35
	ResNet50 V2	99.49	0.05	0.23	97.88	0.23	0.48
	MobileNet V2	66.16	0.60	0.77	97.6	0.08	0.29
	Xception	72.46	0.59	0.77	98.16	0.04	0.20
	DenseNet 169	83.49	0.46	0.67	97.46	0.04	0.20
	EfficientNet B0	95.25	0.59	0.77	98.59	0.05	0.22
	InceptionResNetV2	99.29	0.79	0.89	99.59	0.01	0.11
Leukocyte_400X_915	VGG19	98.59	0.03	0.17	76.28	1.49	1.22
	Inception V3	97.26	0.06	0.24	54.40	2.15	1.46
	EfficientNet B3	99.46	0.02	0.14	96.88	0.59	0.76
	ResNet152 V2	98.49	0.05	0.22	86.32	0.59	0.77
	ResNet50 V2	98.59	0.05	0.22	96.88	0.59	0.76
	MobileNet V2	67.26	0.60	0.77	96.6	0.49	0.70
	Xception	72.79	0.59	0.76	99.16	0.26	0.51
	DenseNet 169	83.49	0.46	0.67	94.46	0.56	0.74
	EfficientNet B0	95.21	0.59	0.76	96.59	0.59	0.77
	InceptionResNetV2	99.29	0.79	0.88	99.59	0.59	0.76
RBCs_8995	VGG19	95.84	0.05	0.24	75.26	1.35	1.16
	Inception V3	96.76	0.04	0.21	59.59	2.12	1.45
	EfficientNet B3	97.14	0.05	0.24	98.49	0.04	0.21
	ResNet152 V2	99.33	0.06	0.25	86.59	0.15	0.39
	ResNet50 V2	94.35	0.05	0.24	97.26	0.25	0.50
	MobileNet V2	66.56	0.65	0.80	97.66	0.04	0.21
	Xception	75.76	0.59	0.76	98.15	0.04	0.22
	DenseNet 169	89.58	0.34	0.58	97.59	0.03	0.17
	EfficientNet B0	94.14	0.19	0.43	99.26	0.46	0.68
	InceptionResNetV2	99.38	0.89	0.94	98.49	0.04	0.22
Toxoplasma_400X_3758	VGG19	98.84	0.03	0.19	95.75	0.95	0.97
	Inception V3	97.76	0.06	0.26	94.59	0.34	0.58
	EfficientNet B3	99.14	0.02	0.16	99.59	0.22	0.47
	ResNet152 V2	98.33	0.05	0.22	93.48	0.25	0.50
	ResNet50 V2	98.35	0.05	0.23	98.58	0.24	0.49
	MobileNet V2	67.56	0.60	0.77	94.68	0.25	0.50
	Xception	72.76	0.55	0.74	95.75	0.94	0.97
	DenseNet 169	83.58	0.37	0.60	98.54	0.26	0.50
	EfficientNet B0	95.14	0.10	0.31	99.56	0.49	0.70
	InceptionResNetV2	99.38	0.01	0.1	95.46	0.55	0.74
Trypanosome_2385	VGG19	80.40	0.57	0.75	98.84	0.73	0.85
	Inception V3	99.24	0.02	0.15	99.76	0.80	0.89
	EfficientNet B3	99.32	0.02	0.16	91.14	0.83	0.91
	ResNet152 V2	99.45	0.02	0.14	96.33	0.14	0.37
	ResNet50 V2	99.91	0.003	0.05	98.35	0.47	0.68
	MobileNet V2	99.59	0.95	0.97	71.56	0.40	0.63
	Xception	94.46	0.34	0.58	75.76	0.35	0.59
	DenseNet 169	99.59	0.25	0.50	87.58	0.17	0.41
	EfficientNet B0	93.76	0.24	0.49	99.14	0.04	0.2
	InceptionResNetV2	91.29	0.22	0.47	94.38	0.01	0.1
Leukocyte_1000X_461	VGG19	98.84	0.73	0.85	99.75	0.95	0.97
	Inception V3	94.76	0.80	0.89	94.59	0.35	0.59
	EfficientNet B3	91.14	0.83	0.91	99.56	0.25	0.50
	ResNet152 V2	95.45	0.59	0.77	93.86	0.47	0.69
	ResNet50 V2	95.75	0.75	0.86	95.45	0.29	0.54
	MobileNet V2	96.91	0.53	0.73	95.85	0.17	0.41
	Xception	96.45	0.59	0.76	97.94	0.24	0.49
	DenseNet 169	91.35	0.75	0.87	90.45	0.18	0.43
	EfficientNet B0	99.75	0.59	0.76	95.56	0.17	0.41
	InceptionResNetV2	98.84	0.73	0.85	99.75	0.95	0.97
Plasmodium_843	VGG19	98.84	0.73	0.85	98.84	0.73	0.85
	Inception V3	94.76	0.80	0.89	94.76	0.80	0.89
	EfficientNet B3	91.14	0.83	0.91	80.61	0.59	0.76
	ResNet152 V2	96.33	0.14	0.37	99.73	0.008	0.08
	ResNet50 V2	98.35	0.47	0.68	99.52	0.02	0.15
	MobileNet V2	98.75	0.86	0.92	99.73	0.008	0.08
	Xception	94.95	0.59	0.77	99.95	0.001	0.03
	DenseNet 169	97.85	0.75	0.86	80.61	0.59	0.76
	EfficientNet B0	96.34	0.58	0.76	98.22	0.59	0.76
	InceptionResNetV2	91.49	0.86	0.92	94.46	0.46	0.67
Toxoplasma_1000X_2933	VGG19	86.28	0.59	0.77	99.75	0.95	0.97
	Inception V3	98.40	0.57	0.75	94.59	0.35	0.59
	EfficientNet B3	97.88	0.56	0.74	99.56	0.25	0.50
	ResNet152 V2	91.32	0.46	0.68	93.48	0.25	0.50
	ResNet50 V2	90.88	0.48	0.69	99.58	0.20	0.44
	MobileNet V2	90.86	0.86	0.93	99.68	0.23	0.48
	Xception	98.84	0.73	0.85	91.75	0.95	0.97
	DenseNet 169	94.76	0.80	0.89	98.54	0.29	0.53
	EfficientNet B0	91.14	0.83	0.91	94.59	0.46	0.67
	InceptionResNetV2	96.33	0.14	0.37	91.46	0.59	0.76
Trichomonad_10134	VGG19	99.78	0.10	0.31	98.97	0.12	0.35
	Inception V3	85.62	505.75	22.48	98.49	0.10	0.31
	EfficientNet B3	94.84	7.25	2.69	98.89	0.84	0.92
	ResNet152 V2	99.50	0.09	0.30	78.27	975.80	31.2
	ResNet50 V2	95.13	13.96	3.73	93.88	10.18	3.19
	MobileNet V2	98.97	0.12	0.35	99.97	0.26	0.51
	Xception	99.49	0.10	0.31	92.01	23.63	4.86
	DenseNet 169	93.38	39.62	6.29	97.31	0.52	0.72
	EfficientNet B0	97.31	0.52	0.72	99.01	0.22	0.47
	InceptionResNetV2	99.01	0.22	0.47	94.53	36.62	6.05

The table analyses the performance of 10 different deep learning models on 10 different classes of datasets based on their accuracy, loss, and RMSE. InceptionResNetV2 performs very well on the Leishmania, Babesia, and Leukocyte datasets, but it does not perform well on the Trypanosome dataset. EfficientNet B3 and Resnet50V2 perform well on most of the datasets except the Plasmodium and Toxoplasma datasets respectively. Overall, InceptionResNetV2 is the best-performing model on most of the datasets. MobileNet V2 computed the lowest accuracy on the Trypanosome dataset but achieved the highest one on the Trichomonad dataset. This signifies that MobileNet V2 is a good choice for tasks where the dataset is small or complex. Xception has the lowest accuracy on the Trypanosome dataset and the Toxoplasma dataset, but it has the highest accuracy on the Plasmodium dataset. Likewise, DenseNet 169 as well as InceptionV3 have the lowest accuracies on most of the datasets.

**Figure 11:**
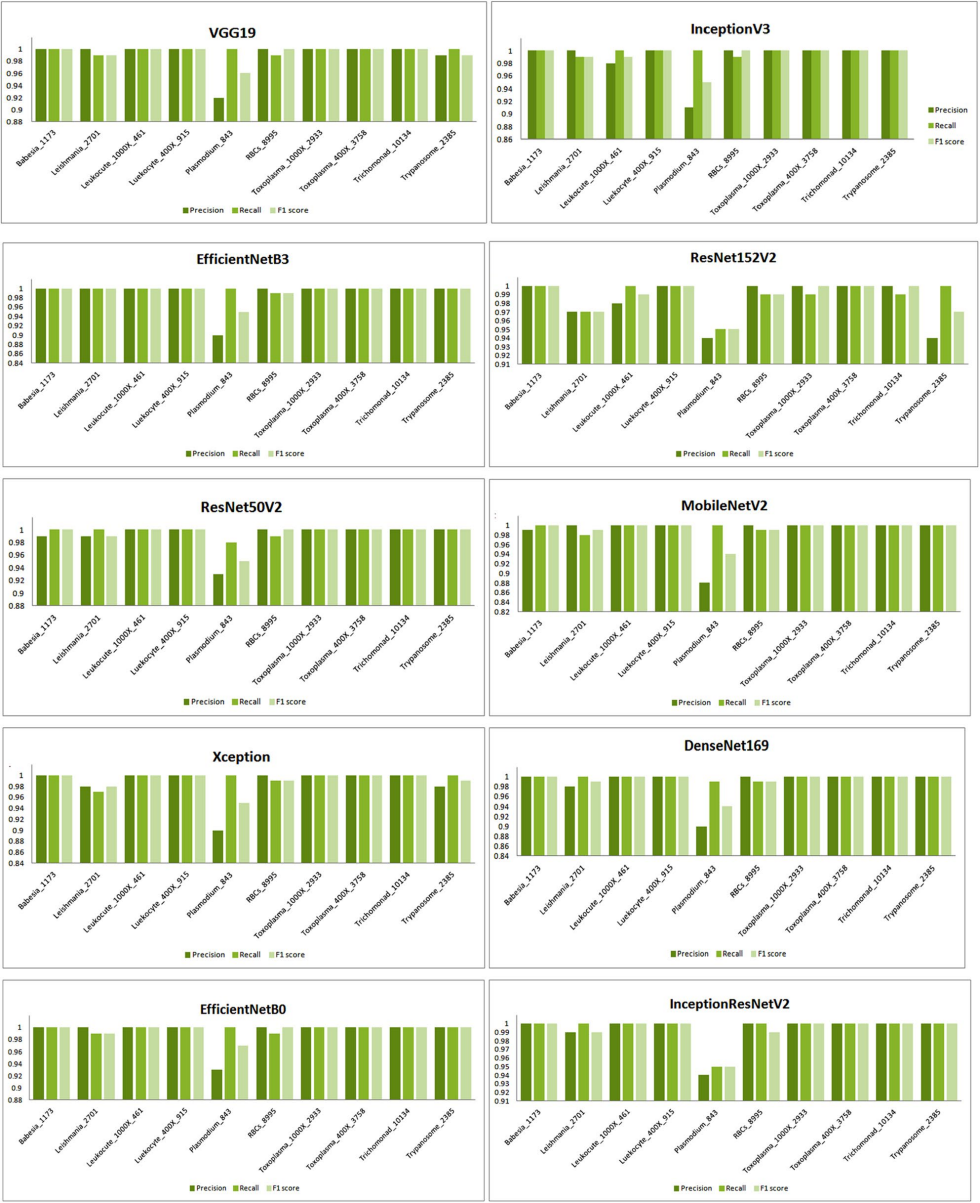
Examining the performance of models for different classes using SGD.

Besides this, the performance of the models has been also analyzed by using different performance metrics such as precision, recall, and F1 score for different classes of parasites as shown in Fig. 11.

### Adam

This subsection defines the performance of the models for different performance metrics on fine tuning their parameters by using ADAM optimizer.

On analyzing the models, it can be seen that all models did quite well during training as well as the validation period, as shown in Table 9. It has been found that InceptionResNetV2, EfficientNetB3, and VGG19 performed well by computing 99.99%, 99.98%, and 99.92% training accuracies along with the lowest loss and RMSE values of 0.12 (0.34), 0.17 (0.41), and 0.16 (0.40) respectively as compared to the others. On the contrary, it has been found that for the validation phase, InceptionResNetV2computed the highest accuracy of 99.96% followed by InceptionV3 and EfficientNetB3 obtained an accuracy of 99.94% and 99.91% respectively while the best loss and RMSE values have been obtained by InceptionResNetV2 with 0.13(0.36) followed by EfficientNetB0 and VGG19 with 0.14(0.37) each.

**Table 9:** Evaluation of models during training and validation phases using Adam optimizer.

Models	Training records			Validation records		
	Accuracy	Loss	RMSE Value	Accuracy	Loss	rmse value
VGG19	99.92	0.16	0.4	99.85	0.14	0.37
Inception V3	99.85	0.18	0.42	99.94	0.16	0.4
EfficientNet B3	99.98	0.17	0.41	99.91	0.15	0.38
ResNet152 V2	99.79	0.20	0.44	99.59	0.18	0.42
ResNet50 V2	99.45	0.23	0.47	99.35	0.22	0.46
MobileNet V2	99.73	0.24	0.48	99.85	0.21	0.45
Xception	98.60	0.27	0.51	99.25	0.26	0.51
DenseNet 169	99.80	0.19	0.43	99.79	0.16	0.4
EfficientNet B0	99.87	0.16	0.4	99.88	0.14	0.37
Hybrid (InceptionResNetV2)	99.99	0.12	0.34	99.96	0.13	0.36

**Figure 12:**
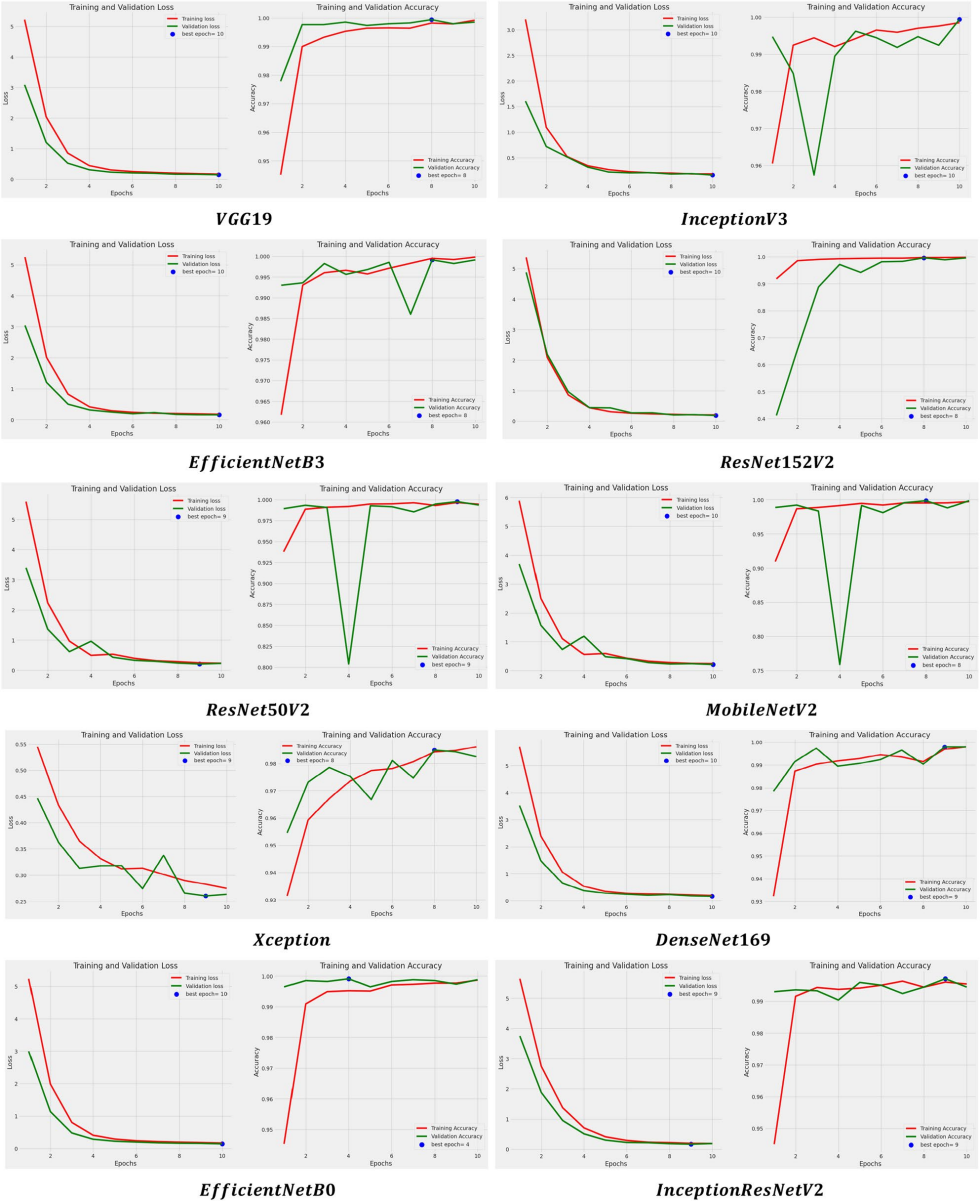
Learning curves of applied models using Adam optimizer.

In addition to this, the models are examined based on their learning curves of accuracy and loss during both training and validation periods as shown in Fig. 12.

The layers of all the models have been iterated for 10 epochs and it can be found that they have obtained their best value or score either at the 10th epoch or in between the 8th to 10th epoch for both accuracy and loss. Besides this, here also large gaps have been seen in the performance of a few models such as MobileNetV2 and ResNet50V2 which directs us toward the overfitting error of the model.

Other than this, the models are also evaluated for another set of parameters i.e. precision, recall, and F1 score whose results are shown in Table 10.

**Table 10:** Analysis of models for different parameters using Adam optimizer.

Models	Precision	Recall	F1 score
VGG19	0.99	1.00	1.00
Inception V3	0.99	1.00	0.99
EfficientNet B3	0.99	1.00	1.00
ResNet152 V2	0.99	1.00	1.00
ResNet50 V2	0.99	1.00	0.99
MobileNet V2	0.99	1.00	0.99
Xception	0.99	1.00	0.99
DenseNet 169	0.97	0.98	0.97
EfficientNet B0	0.99	0.99	0.99
InceptionResNetV2	0.98	0.99	0.98

It can be assayed from the table that the best performance has been showcased by VGG19, EffcieintNetB3, and ResNet152V2 with 0.99, 1.00, and 1.00 as precision, recall, and F1 score respectively followed by InceptionV3, ResNet50V2, MobileNetV2, and Xception. The performance of these models indicates that they perform very well in the classification task, achieving near-perfect accuracy and completeness. On the contrary, DenseNet169 has the lowest precision, recall, and F1 score among the listed models, indicating that it may have more false positives and false negatives compared to the other models. The models other than the mentioned ones also tried their best to perform well for these metrics.

**Figure 13:**
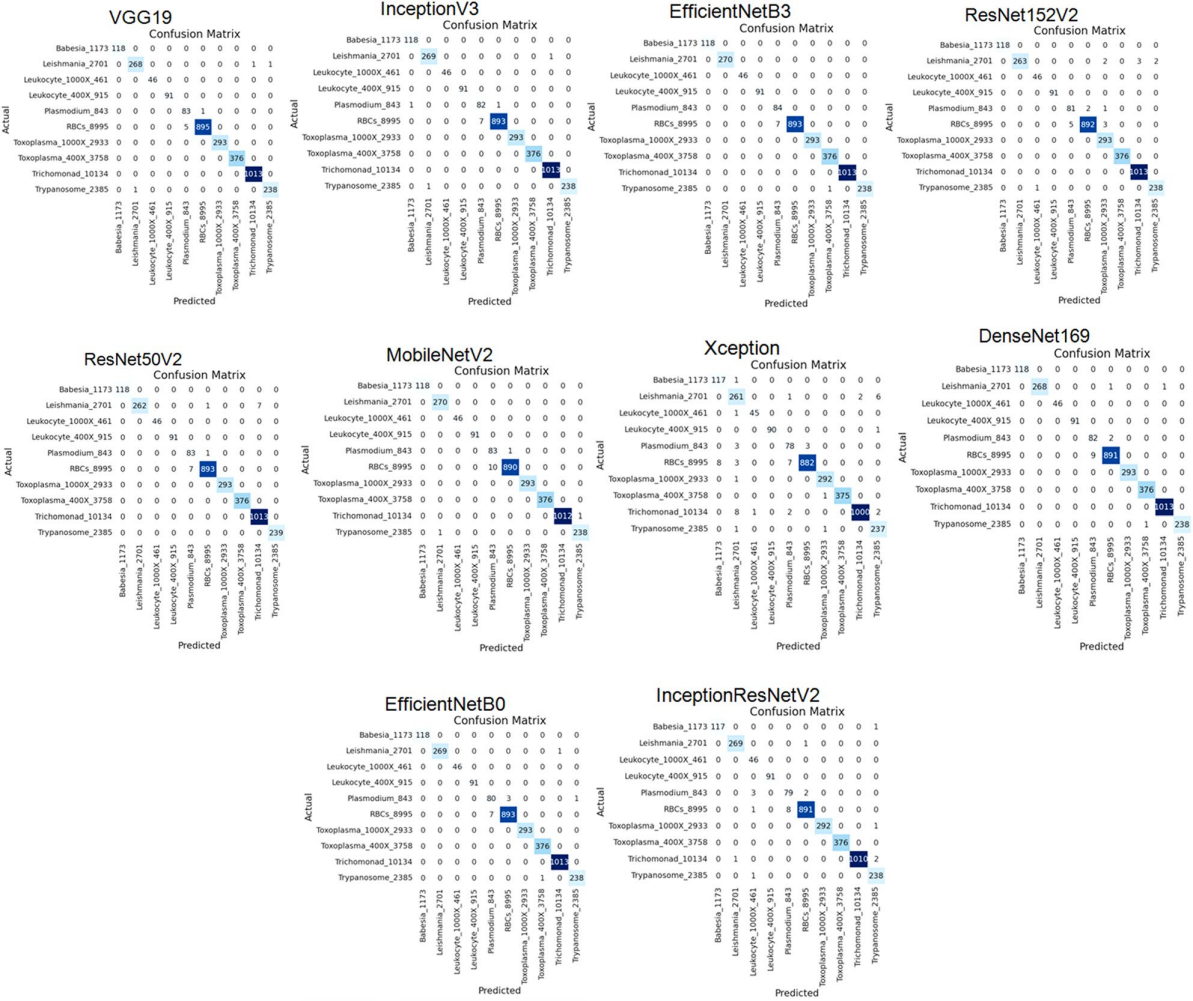
Confusion matrix of applied models uaing Adam optimizer.

After examining the performance of the models for the classification of various parasites, confusion matrices of 10 × 10 have been generated which is a crucial tool for assessing the performance of multi-class classification models, as shown in Fig. 13. Based on these matrices, true positive, true negative, false positive, and false negative values are being taken to examine the performance of models for different classes, as shown in Table 11.

**Table 11:** Evaluation of models during training and validation phases for different classes using Adam optimizer.

Classes	Models	Training Records			Validation Records		
		Accuracy	Loss	RMSE	Accuracy	Loss	RMSE
Babesia_1173	VGG19	98.40	0.39	0.62	94.59	3.50	1.87
	Inception V3	99.29	0.28	0.52	96.49	0.89	0.94
	EfficientNet B3	98.46	0.02	0.14	98.59	0.35	0.59
	ResNet152 V2	97.31	0.34	0.59	99.56	0.24	0.49
	ResNet50 V2	62.63	159.46	12.6	99.76	0.04	0.22
	MobileNet V2	94.59	3.50	1.87	98.49	0.37	0.61
	Xception	96.49	0.89	0.94	54.21	343.04	18.5
	DenseNet 169	99.00	0.15	0.39	88.67	2.36	1.53
	EfficientNet B0	99.56	0.24	0.49	98.59	0.49	0.7
	InceptionResNetV2	99.76	0.04	0.22	96.36	2.89	1.7
Leishmania_2701	VGG19	98.59	0.35	0.59	96.49	0.34	0.58
	Inception V3	97.56	0.24	0.49	62.29	149.59	12.2
	EfficientNet B3	98.76	0.05	0.24	90.31	3.90	1.97
	ResNet152 V2	96.49	0.34	0.58	94.49	2.79	1.67
	ResNet50 V2	62.29	149.59	12.2	97.26	0.35	0.59
	MobileNet V2	94.46	3.04	1.74	98.59	4.022	2.00
	Xception	98.76	0.84	0.92	56.21	043.04	6.56
	DenseNet 169	99.49	0.14	0.38	87.67	2.59	1.60
	EfficientNet B0	87.67	2.59	1.60	96.59	0.86	0.92
	InceptionResNetV2	96.59	0.86	0.92	98.36	2.59	1.60
Leukocyte_400X_915	VGG19	95.46	3.04	1.74	94.49	3.00	1.73
	Inception V3	99.76	0.85	0.92	96.55	0.89	0.94
	EfficientNet B3	98.59	0.35	0.59	98.52	0.34	0.59
	ResNet152 V2	99.56	0.24	0.49	99.29	0.76	0.87
	ResNet50 V2	99.76	0.04	0.22	98.76	0.49	0.70
	MobileNet V2	98.49	0.37	0.61	97.19	0.35	0.59
	Xception	64.29	149.35	12.2	62.46	159.40	12.6
	DenseNet 169	95.46	3.04	1.74	94.49	3.00	1.73
	EfficientNet B0	99.76	0.85	0.92	96.55	0.89	0.94
	InceptionResNetV2	99.49	0.17	0.41	99.29	0.11	0.33
RBCs_8995	VGG19	97.63	0.87	0.93	88.59	2.29	1.51
	Inception V3	97.45	0.34	0.59	98.22	0.48	0.69
	EfficientNet B3	94.05	0.27	0.52	91.49	4.94	2.22
	ResNet152 V2	99.36	0.04	0.21	95.46	1.77	1.33
	ResNet50 V2	94.38	0.31	0.56	98.19	0.31	0.56
	MobileNet V2	66.63	149.73	12.2	96.46	1.00	1
	Xception	98.63	3.04	1.74	54.76	300.94	17.3
	DenseNet 169	97.63	0.87	0.93	88.59	2.29	1.51
	EfficientNet B0	96.67	0.12	0.35	98.22	0.48	0.69
	InceptionResNetV2	98.63	3.04	1.74	96.76	2.84	1.68
Toxoplasma_400X_3758	VGG19	98.59	0.49	0.7	91.31	4.90	2.21
	Inception V3	96.36	2.89	1.7	95.82	1.79	1.33
	EfficientNet B3	98.45	0.39	0.62	98.23	0.35	0.59
	ResNet152 V2	99.05	0.28	0.52	96.36	1.02	1.01
	ResNet50 V2	99.36	0.08	0.29	54.21	343.04	18.5
	MobileNet V2	98.38	0.31	0.56	88.67	2.36	1.53
	Xception	64.63	149.33	12.2	98.59	0.49	0.7
	DenseNet 169	95.63	3.09	1.75	96.36	2.89	1.7
	EfficientNet B0	99.63	0.89	0.94	91.31	4.90	2.21
	InceptionResNetV2	99.67	0.13	0.37	95.82	1.79	1.33
Trypanosome_2385	VGG19	99.49	0.15	0.39	98.89	0.84	0.92
	Inception V3	85.56	500.45	22.3	78.27	975.80	31.2
	EfficientNet B3	94.29	6.24	2.49	93.88	10.18	3.19
	ResNet152 V2	99.76	0.04	0.21	98.97	0.26	0.51
	ResNet50 V2	95.00	11.79	3.43	92.01	23.63	4.86
	MobileNet V2	98.49	1.12	1.06	97.31	0.52	0.72
	Xception	99.49	1.10	1.04	99.01	0.22	0.47
	DenseNet 169	93.59	35.62	5.96	94.53	36.62	6.05
	EfficientNet B0	99.49	0.15	0.39	98.89	0.84	0.92
	InceptionResNetV2	85.56	500.45	22.30	78.27	975.80	31.2
Leukocyte_1000X_461	VGG19	99.49	0.15	0.39	98.73	0.49	0.70
	Inception V3	85.56	505.46	22.42	94.59	12.79	3.57
	EfficientNet B3	94.29	7.27	2.69	98.49	0.86	0.92
	ResNet152 V2	99.76	0.05	0.23	84.49	500.49	22.3
	ResNet50 V2	95.00	13.76	3.71	93.52	6.67	2.58
	MobileNet V2	98.49	0.15	0.39	98.73	0.49	0.70
	Xception	99.49	0.50	0.70	94.59	12.79	3.57
	DenseNet 169	93.59	39.00	6.24	97.46	0.34	0.58
	EfficientNet B0	98.49	0.15	0.39	98.90	0.20	0.44
	InceptionResNetV2	99.49	0.50	0.70	92.34	38.66	6.21
Plasmodium_843	VGG19	99.78	0.11	0.33	99.49	0.20	0.44
	Inception V3	85.62	500.75	22.3	99.46	0.15	0.39
	EfficientNet B3	94.84	6.25	2.50	85.76	505.46	22.4
	ResNet152 V2	99.50	0.19	0.44	94.00	7.27	2.69
	ResNet50 V2	95.13	12.96	3.60	99.49	0.05	0.23
	MobileNet V2	98.97	0.22	0.47	95.76	13.76	3.71
	Xception	99.49	0.20	0.44	98.59	0.15	0.39
	DenseNet 169	93.38	38.62	6.21	99.76	0.50	0.70
	EfficientNet B0	99.78	0.11	0.33	93.59	39.00	6.24
	InceptionResNetV2	99.76	0.50	0.70	99.46	0.15	0.39
Toxoplasma_1000X_2933	VGG19	95.89	0.84	0.92	92.31	0.52	0.72
	Inception V3	75.49	975.80	31.2	95.01	0.22	0.47
	EfficientNet B3	90.76	10.18	3.19	99.49	0.15	0.39
	ResNet152 V2	96.59	0.26	0.51	85.46	505.49	22.4
	ResNet50 V2	90.46	23.63	4.86	94.28	7.27	2.69
	MobileNet V2	92.31	0.52	0.72	99.55	0.05	0.23
	Xception	95.01	0.22	0.47	95.76	13.49	3.67
	DenseNet 169	90.53	36.62	6.05	98.26	0.17	0.42
	EfficientNet B0	95.01	0.22	0.47	99.76	0.15	0.39
	InceptionResNetV2	99.49	0.15	0.39	93.00	39.46	6.28
Trichomonad_10134	VGG19	98.49	0.86	0.92	90.76	10.14	3.18
	Inception V3	84.49	500.49	22.3	96.59	0.25	0.50
	EfficientNet B3	93.52	6.67	2.58	95.89	0.84	0.92
	ResNet152 V2	98.73	0.49	0.70	75.49	975.49	31.2
	ResNet50 V2	94.59	12.79	3.57	90.76	10.14	3.18
	MobileNet V2	97.46	0.34	0.58	96.59	0.25	0.50
	Xception	98.90	0.20	0.44	90.46	23.46	4.84
	DenseNet 169	92.34	38.66	6.21	92.31	0.55	0.74
	EfficientNet B0	97.46	0.34	0.58	95.01	0.22	0.47
	InceptionResNetV2	98.90	0.20	0.44	90.53	36.62	6.05

In the case of Babesia_1173, we can observe that InceptionResNetV2 and EfficientNetB0 achieved impressively high training accuracies of 99.76% and 99.56%, respectively. However, Xception experienced a noticeable drop in validation accuracy to 54.21%, indicating some level of overfitting from 96.49% accuracy during the training phase. EfficientNet B3 showed consistency between training and validation with accuracies of 98.46% and 98.59%, respectively, suggesting its stability. In contrast, ResNet50 V2 exhibited lower training accuracy (62.63%) but excelled on the validation set with 99.76% accuracy, indicating the potential for generalization despite challenges in the training phase. Moving to Leishmania_2701, we observe VGG19 and Inception V3 performing well in training with accuracies of 98.59% and 97.56%, respectively. However, Inception V3 and Xception struggled to generalize, achieving only 62.29% and 56.21% accuracy respectively on validation. EfficientNet B3 showcased consistency with training and validation accuracies of 98.76% and 90.31%, respectively. ResNet50 V2's training accuracy was lower (62.29%), but its validation performance was strong at 97.26%. In the case of RBCs_8995, VGG19, and xception had a noticeable gap between training (97.63%) and validation (88.59%) accuracy and training (98.73) as well as validation (54.76%) accuracy respectively, suggesting overfitting. In contrast, Inception V3 exhibited strong performance with 97.45% training and 98.22% validation accuracy. EfficientNet B3 and DesneNet169 faced challenges with 94.05% and 97.63% training and 91.49% and 88.59% validation accuracy respectively. ResNet152 V2 showcased robustness with 99.36% training and 95.46% validation accuracy, while ResNet50 V2 demonstrated consistent performance with 94.38% training and 98.19% validation accuracy. Lastly, for Toxoplasma_400X_3758, InceptionresNetV2 achieved a high training accuracy of 99.67% and VGG19 as well as EfficientNetB0 obtained a lower validation accuracy of 91.31%. EfficientNet B3 exhibited consistency with training (98.45%) and validation (98.23%) accuracy. ResNet152 V2 excelled with 99.05% training and 96.36% validation accuracy, while Xception faced challenges with 64.63% accuracy and 149.33 loss training but performed well on the validation dataset with 98.59% accuracy. Similarly, ResNet50V2 performed well for the training dataset with 99.36% accuracy and showed the lowest performance at the validation phase with 54.21% and 343 loss.

**Figure 14:**
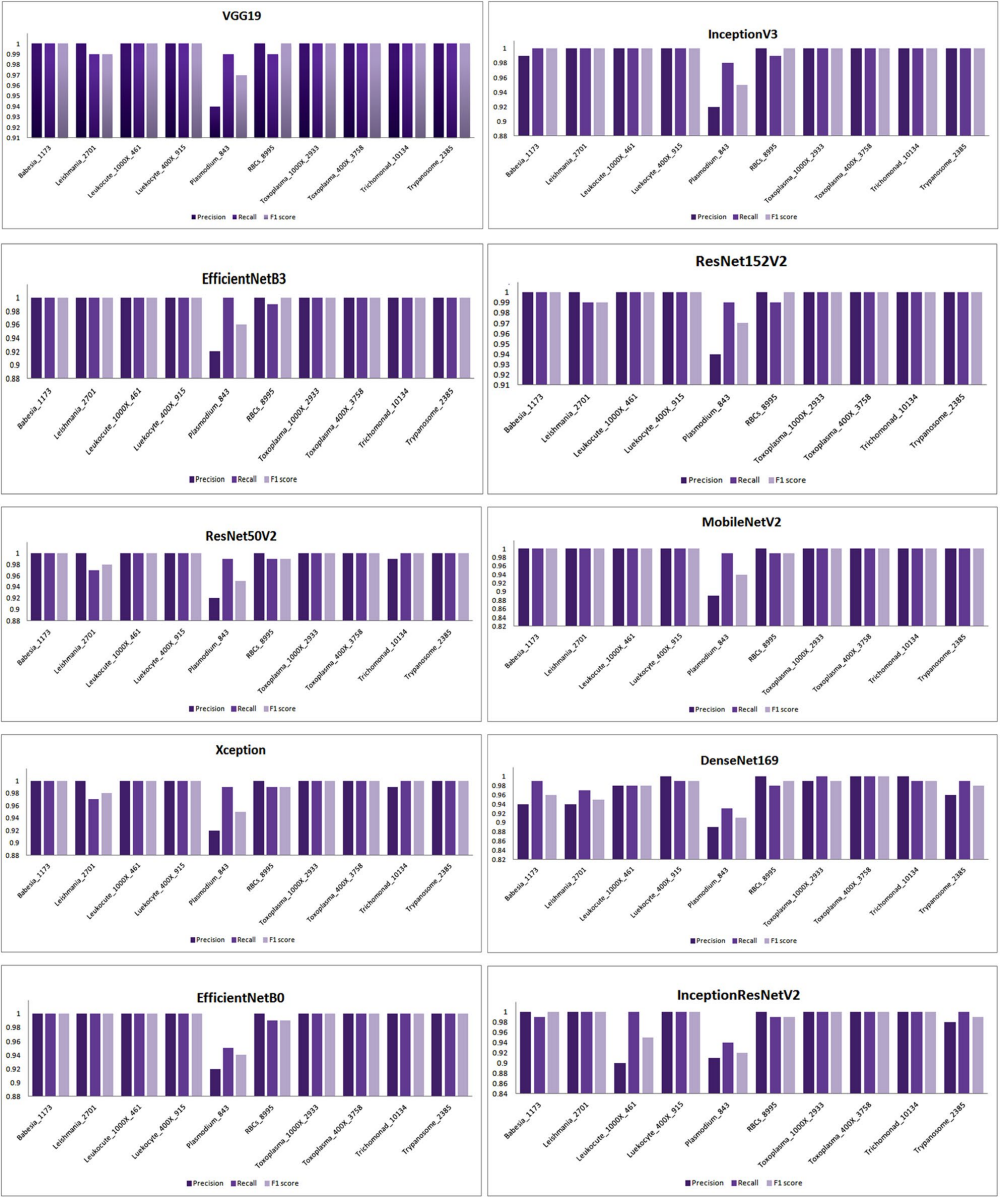
Performance of the models for different classes using Adam optimizer.

In the same way, the performance of models for the other set of classes is also being examined and the results are mentioned in the aforementioned table. Besides this, the performance of the models has been also examined graphically for precision, recall, and F1 score on being trained with a different class of dataset as shown in Fig. 14.

### Computational time

While training the deep learning with the dataset, the computational time varies with various factors as well as the choice of optimizers which plays an important role in this regard. In Table 12, training times of the applied models with different optimizers i.e. RMSprop, SGD, and Adam have shown some interesting behaviours.

**Table 12:** Computational time of the models.

Models	RMSprop	SGD	ADAM
VGG19	6 h 5 min	8 h 50 min	4 h 5 min
Inception V3	10 h	11 h	12 h 12 min
EfficientNet B3	4 h 50 min	6 h 5 min	8 h 5 min
ResNet152 V2	15 h	1 h	10 h
ResNet50 V2	12 h 5 min	14 h 15 min	10 h 46 min
MobileNet V2	6 h 7 min	5 h 17 min	16 h 7 min
Xception	2 h 50 min	4 h 30 min	3 h 51 min
DenseNet 169	11 h 4 min	13 h 14 min	15 h 49 min
EfficientNet B0	4 h 9 min	7 h 8 min	8 h 56 min
Hybrid (InceptionResNetV2)	16 h 20 min	11 h 30 min	12 h 50 min

It has been found that on incorporating the RMSprop optimizer, the Xception model did the training of the dataset within 2 h 50 min while the maximum time was taken by InceptionResNetV2 with 16 h 20 min. Likewise, on using SGD optimizer to fine-tune the parameter of deep learning models, the minimum time to train the dataset was taken by ResNet152V2 with 1 h and the maximum was taken by ResNet50V2 with 14 h 15 min. In the end, fine-tuning the parameters of deep learning with ADAM optimizer, the model which computed the least time is VGG19 with 4 h 5 min and the max was taken by DenseNet169 15 h 49 min.

RMSprop and SGD appear to be the fastest optimizer for most of the models, with shorter training times compared to Adam. This suggests that RMSprop and SGD are efficient at converging to good model weights quickly. However, it's essential to consider that the performance of the optimizer may vary depending on the specific problem, dataset, and hyperparameters. On the other hand, Adam, a popular optimizer known for its adaptive learning rates, often falls between RMSprop and SGD in terms of training times.

## Conclusion

This study represents a significant advancement in the field of parasitic disease detection and classification. Harnessing the capabilities of deep learning models, coupled with meticulous image processing techniques, this research has demonstrated exceptional accuracy and efficiency in identifying and categorizing various parasitic organisms. The integration of deep learning models, including VGG19, InceptionV3, EfficientNetB3, Xception, MobileNetV2, ResNet50V2, ResNet152V2, DenseNet169, EfficientNetB0, and InceptionResNetV2, along with strategic optimization using RMSprop, SGD, and Adam, has yielded remarkable results. Incorporating these optimizers significantly enhanced the performance of the models, with InceptionResNetV2 achieving the highest accuracy.

Furthermore, the applied models were evaluated based on precision, recall, and F1 score, consistently achieving values around 0.99. This research not only demonstrates the effectiveness of artificial intelligence in parasitology but also underscores the importance of interdisciplinary approaches in scientific research. Despite these achievements, certain challenges were encountered, such as overfitting due to large iteration gaps and extended computational time required for training with the dataset. Addressing these challenges in future research is crucial, and diversifying the training dataset with a broader range of parasitic organisms is recommended to enhance the model's robustness and applicability in real-world scenarios.

## Data Availability

The dataset used in the study is publically available at the below link: https://data.mendeley.com/datasets/38jtn4nzs6/2.
